# Microalgae-Derived Proteins for Sustainable Foods and Beverages: Sources, Extraction, Characterization, and Applications

**DOI:** 10.3390/foods15172972

**Published:** 2026-08-24

**Authors:** Elisa Costa, Miguel Ribeiro, Luís Filipe-Ribeiro, Fernanda Cosme, Fernando M. Nunes

**Affiliations:** 1 Chemistry Research Centre of Vila Real (CQ-VR)—Food and Wine Chemistry Laboratory, University of Trás-os-Montes and Alto Douro, 5000-801 Vila Real, Portugal; ecmatos@gmail.com (E.C.); jmribeiro@utad.pt (M.R.); fmota@utad.pt (L.F.-R.); fcosme@utad.pt (F.C.); 2 Genetics and Biotechnology Department, University of Trás-os-Montes and Alto Douro, 5000-801 Vila Real, Portugal; 3 Biology and Environment Department, University of Trás-os-Montes and Alto Douro, 5000-801 Vila Real, Portugal; 4 Chemistry Department, University of Trás-os-Montes and Alto Douro, 5000-801 Vila Real, Portugal

**Keywords:** microalgae protein, *Arthrospira platensis*, *Chlorella vulgaris*, *Tetraselmis chuii*, alternative proteins, protein extraction, protein characterization, non-animal-derived, non-allergenic, sustainable, food applications

## Abstract

The growing demand for sustainable, non-animal-derived, and low-allergenic protein alternatives has driven research into innovative sources such as microalgae. This review focuses on three key microalgal species *Arthrospira platensis* (Spirulina), *Chlorella vulgaris*, and *Tetraselmis chuii*. It examines their cell wall structures, protein content, and amino acid profiles. Protein extraction methods, including physical, enzymatic, and chemical approaches, are critically discussed. Downstream purification techniques aimed at improving protein purity and quality are also reviewed. Protein characterization methods are discussed, highlighting their relevance to food applications. The potential applications of microalgal biomass and protein extracts in food and beverage products are evaluated, with consideration given to their functionality, safety, and regulatory aspects. Despite significant advances in this field, further research is essential to optimize extraction and processing technologies, facilitate their integration into mainstream food production, and improve overall process efficiency.

## 1. Introduction

The extraction of proteins from plant and animal sources has gained significant attention due to the nutritional value and functional properties of these proteins, which play a crucial role in food processing and beverage production [1]. However, identifying and adopting alternative protein sources is essential to meeting growing consumer demands while addressing the environmental and resource-related challenges associated with traditional food production systems [2]. This transition can contribute to environmental sustainability by reducing the ecological footprint of livestock farming, improving resource efficiency, and diversifying protein sources [3].

The term “algae” encompasses a vast range of photosynthetic organisms, categorized by size into two main groups: microalgae (which include both prokaryotic and eukaryotic unicellular organisms or simple colonies) and macroalgae (which are exclusively eukaryotic, and predominantly multicellular) [4]. Microalgae species are highly adaptable organisms, characterized by rapid growth rates, minimal water requirements, limited competition for arable land, and a comparatively low environmental impact [2]. These attributes make microalgae promising candidates for climate change mitigation and the development of more sustainable food systems [5].

The safety of microalgae for human consumption has been extensively studied over the past two decades [4]. In the European Union (EU), microalgae commercialization for food applications is regulated under Regulation (EU) 2015/2283 [6], which governs novel foods. Similarly, the United States Food and Drug Administration (FDA) has classified certain microalgae species as generally recognized as safe (GRAS).

The genus *Arthrospira*, commonly known as Spirulina, includes some of the most widely studied microalgae. The main species are *Arthrospira platensis* (also known as *Spirulina platensis*) and *Arthrospira maxima* (also known as *Spirulina maxima*) [7]. Both species have GRAS status in the United States and are considered non-novel foods in the European Union. They also have a long history of human consumption, particularly in China. Additionally, *Chlorella vulgaris* was granted GRAS status by the FDA in 2013 and is also classified as a non-novel food in the EU, supported by extensive safety data and historical use across diverse cultures. Today, *Chlorella vulgaris* and Spirulina are among the most widely cultivated microalgae species worldwide [8].

More recently, the portfolio of commercially produced microalgal strains has expanded to include species, such as *Tetraselmis chuii*, *Nannochloropsis oculata*, *Scenedesmus obliquus*, *Scenedesmus rubescens*, and *Chlorococcum amblystomatis.* Among these, *Tetraselmis chuii* is classified as a novel food in the EU [9,10]. This review focuses on cyanobacteria *Arthrospira*, a prokaryotic, Gram-negative, organism commonly known as Spirulina, as well as the green microalgae species *Chlorella vulgaris* and *Tetraselmis chuii*.

Beyond their nutritional value, microalgae are increasingly being explored for application across various industries, including biofuel production, bioplastic development, and wastewater treatment [11]. Moreover, their nutritional and health-promoting properties have also garnered significant attention. These include natural pigments used for coloration and bioactive compounds such as polyunsaturated fatty acids, polysaccharides, polyphenols, sterol compounds, minerals, antioxidants, and proteins [12]. Microalgae protein extracts can be incorporated into protein-enriched foods, including cereal-based products, dairy products, beverages, and meat alternatives. This approach may improve the nutritional quality and functionality of these products [13]. In addition, microalgal protein extracts have recently been evaluated as fining agents for use in oenology. A purified Spirulina protein extract, obtained by bead milling followed by ethanol precipitation, was shown to act as an effective protein-based fining agent in model wine systems, removing proanthocyanidins with an efficiency comparable to that of commercial plant- and animal-derived fining agents [14].

In this context, the development of efficient protein extraction and purification techniques is closely linked to advances in biochemical characterization methods. Cell disruption and protein extraction methods include bead milling [15], high-pressure homogenization [16], ultrasound-assisted extraction [3,17], and chemical [18] or enzymatic treatments [15,19,20]. Subsequent separation and purification techniques, such as salt and organic solvent precipitation, as well as dialysis [21], have also been widely explored. Protein characterization methods, including chromatography [22], electrophoresis [23], and proteomic analysis [24], provide further insights into the protein composition, structure, and functionality of extracted proteins. Each method has specific advantages and limitations. The optimal technique depends on factors such as the microalgal species, cell wall characteristics, target proteins, and desired extraction efficiency.

Although several recent reviews have addressed microalgal proteins and their extraction processes [25,26,27,28,29], the present review specifically focuses on protein extraction from microalgae for food and beverage applications. It highlights the potential of microalgal species such as Spirulina (*Arthrospira platensis*), *Chlorella vulgaris*, and *Tetraselmis chuii*. The review focuses on protein extraction, separation, and purification techniques, with an emphasis on optimizing protein recovery, purity, and process scalability. Additionally, it explores the chemical attributes of microalgal protein extracts and their potential applications in the food and beverage industry, addressing the growing demand for sustainable non-animal-derived, and low-allergenic protein sources. To provide a coherent framework for this analysis, the review is structured around a central conceptual framework: cell wall structure dictates the extraction strategy, extraction conditions shape protein properties, and protein properties ultimately guide their suitability for specific food applications.

## 2. Overview of Microalgae

### 2.1. Microalgae Species

Microalgae comprise a highly diverse group of photosynthetic microorganisms that can be broadly classified into prokaryotic organisms (e.g., cyanobacteria such as Spirulina) and eukaryotic microalgae, which are distributed across several taxonomic groups, including the major groups Protozoa, Chromista, and Archaeplastida [30].

**Figure 1:**
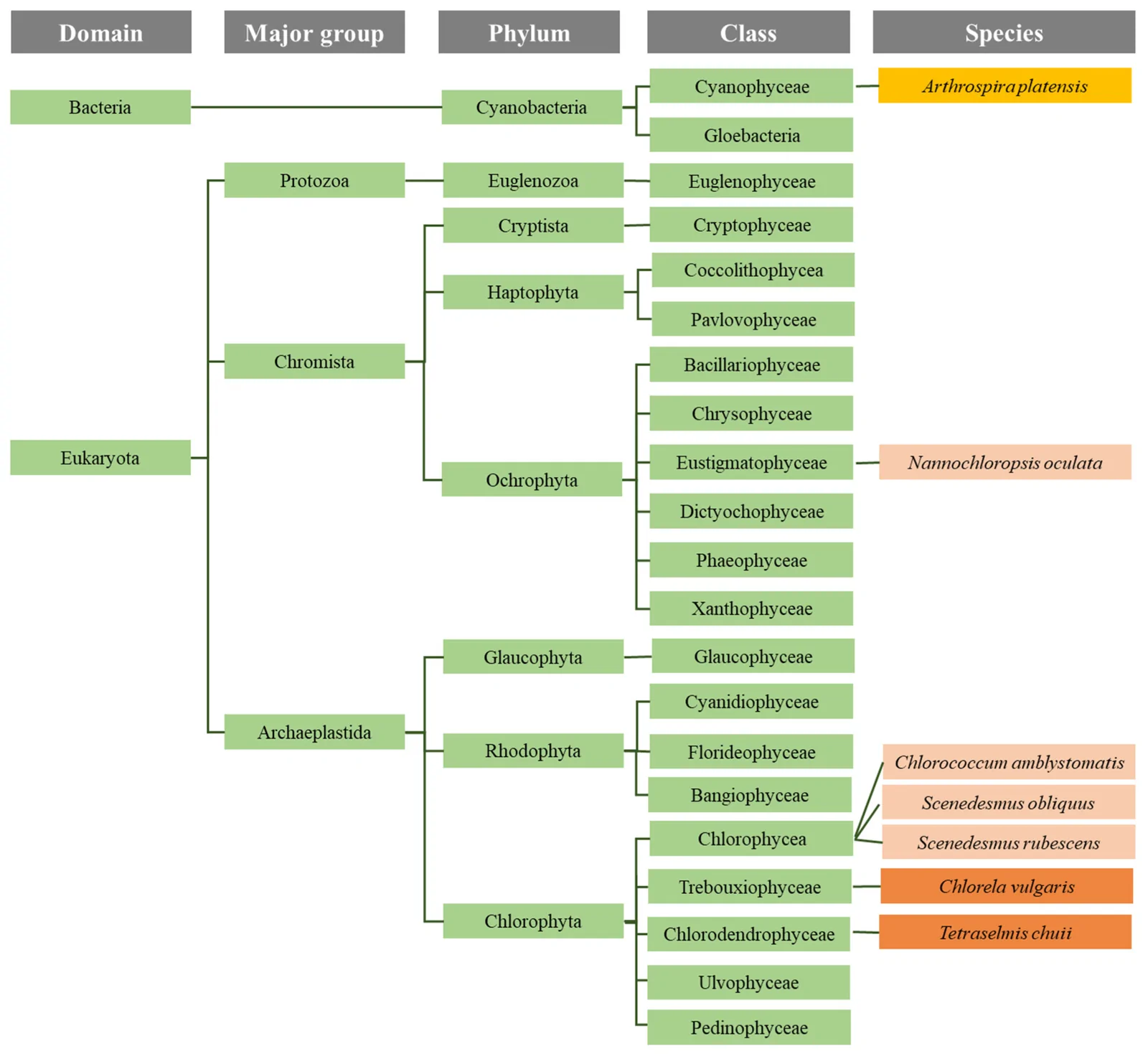
Simplified taxonomic classification of selected algal and cyanobacterial species. Colors are used for visual distinction only and do not convey any additional taxonomic information. Adapted from Guiry [32] and updated according to AlgaeBase.

Prokaryotic *Arthrospira* species belong to the phylum Cyanophyta and are characterized by relatively simple cellular structures and highly efficient photosynthetic capabilities [13]. Eukaryotic microalgae include several well-studied taxonomic groups, such as Chlorophyta (green algae) and other phyla, each exhibiting distinct biological characteristics and ecological functions [17] (Figure 1). Chlorophyta subdivision into classes such as Chlorophyceae, Chlorodendrophyceae, Ulvophyceae, and Pedinophyceae has been substantially revised in recent years following the advent of molecular phylogeny, replacing earlier classifications based solely on morphological and ultrastructural criteria [31].

Despite the vast diversity of microalgal species, estimated at approximately 72,500, only a limited number have been extensively studied. To date, approximately 44,000 species have been formally identified [13]. Furthermore, only a small subset of these species is cultivated and produced commercially, with around 20 species currently being utilized on a commercial scale [2,13]. In practice, commercial exploitation remains concentrated in a few species, such as *Chlorella vulgaris* and *Arthrospira platensis,* which are widely used as food ingredients, human nutritional supplements, and animal feed [8].

The cultivation of microalgae offers several advantages, including minimal land use requirements, relatively low nutrient demands, year-round production, and greater sustainability compared with traditional agricultural systems [5]. Growth conditions can be optimized to maximize productivity by adjusting factors such as light intensity, temperature, and nutrient availability [2]. The cultivation medium is a key control point for both biomass growth and metabolic output: as changes in nutrient supply can shift biomass accumulation and redirect carbon flow toward proteins, lipids, pigments, or other target biomolecules [26] However, these same cultivation variables also contribute to the large inter- and intra-species variability reported in microalgal biomass composition. Thus, protein content should not be interpreted as an intrinsic and constant characteristic of a species, but rather as a variable trait influenced by genotype, cultivation conditions, and harvest stage. This variability is further compounded by taxonomic instability within several commercially relevant genera: species once grouped under a single name have frequently been reassigned following molecular phylogenetic revision, meaning that earlier compositional data may correspond to a taxonomically distinct organism. This taxonomic inconsistency can complicate direct comparisons across studies [31].

Microalgae can be cultivated in open systems, such as pond lagoons, paddlewheel raceways, and cascading raceways, as well as in closed systems, including vertical or horizontal tubular photobioreactors, flat-panel reactors, and other specialized configurations [2]. Open ponds generally have large surface areas and shallow depths and are primarily operated outdoors [33]. These systems offer advantages such as minimal capital investment and relatively simple operation. However, they are also prone to contamination and fluctuations in environmental conditions and provide limited control over cultivation parameters [34]. In contrast, photobioreactors are enclosed systems designed to provide a more controlled environment for microalgal cultivation, enabling more precise regulation of growth conditions [35].

The biochemical composition of microalgae varies considerably among species and is strongly influenced by cultivation conditions. Reported compositions range from 9–77% protein, 6–54% carbohydrates, and 4–74% lipids, depending on the species and cultivation conditions [4]. Notable examples include Spirulina, which can contain up to 77% protein, *Chlorella vulgaris* with a protein content up to 53%, and *Tetraselmis* sp., which exhibits a protein content ranging from 14–58% [36]. Proteins derived from microalgae are often of high nutritional quality, containing all essential amino acids in proportions comparable to those of conventional animal-derived proteins [37].

Several emerging microalgae species have also demonstrated considerable nutritional potential. *Nannochloropsis oculata* (Eustigmatophyceae), for example, is primarily valued for its high lipid content (25–45% dw), particularly eicosapentaenoic acid, but it also possesses a protein profile enriched in essential amino acids (41.5% of total amino acids), making it suitable for nutritional applications [38]. Similarly, *Scenedesmus obliquus*, and *Scenedesmus rubescens* (Chlorophyceae) have been identified as promising candidates due to their high protein content (40.7% and 31.0% dw, respectively) and balanced amino acid profiles, demonstrating resilience under diverse environmental conditions, supporting their potential for sustainable production [39]. Although comparatively less studied, *Chlorococcum amblystomatis* (Chlorophyceae), has also emerged as a potential alternative protein source, warranting further investigation (Figure 1).

In addition to proteins, microalgae are valuable sources of numerous bioactive compounds. They provide lipids rich in omega-3 and omega-6 fatty acids, carbohydrates in the form of polysaccharides, and bioactive pigments such as carotenoids and chlorophylls, enhancing their versatility for functional food and health-related applications [40].

Spirulina is particularly rich in phycobiliproteins, including phycocyanin, while *Chlorella* contains abundant chlorophyll and carotenoids like astaxanthin and lutein [41]. The chlorophyll content in *Chlorella* is approximately 7% of its biomass, which is about five times greater than that reported for Spirulina [42]. *Tetraselmis* exhibits a combination of chlorophyll-a and chlorophyll-b, as well as carotenoids, such as astaxanthin and β-carotene [43].

Efficient post-harvest processes, such as dewatering, drying, and protein extraction, are critical for preserving the nutritional and functional properties of microalgal biomass [33].

### 2.2. Structural Features: Cell Wall of Microalgae

**Figure 2:**
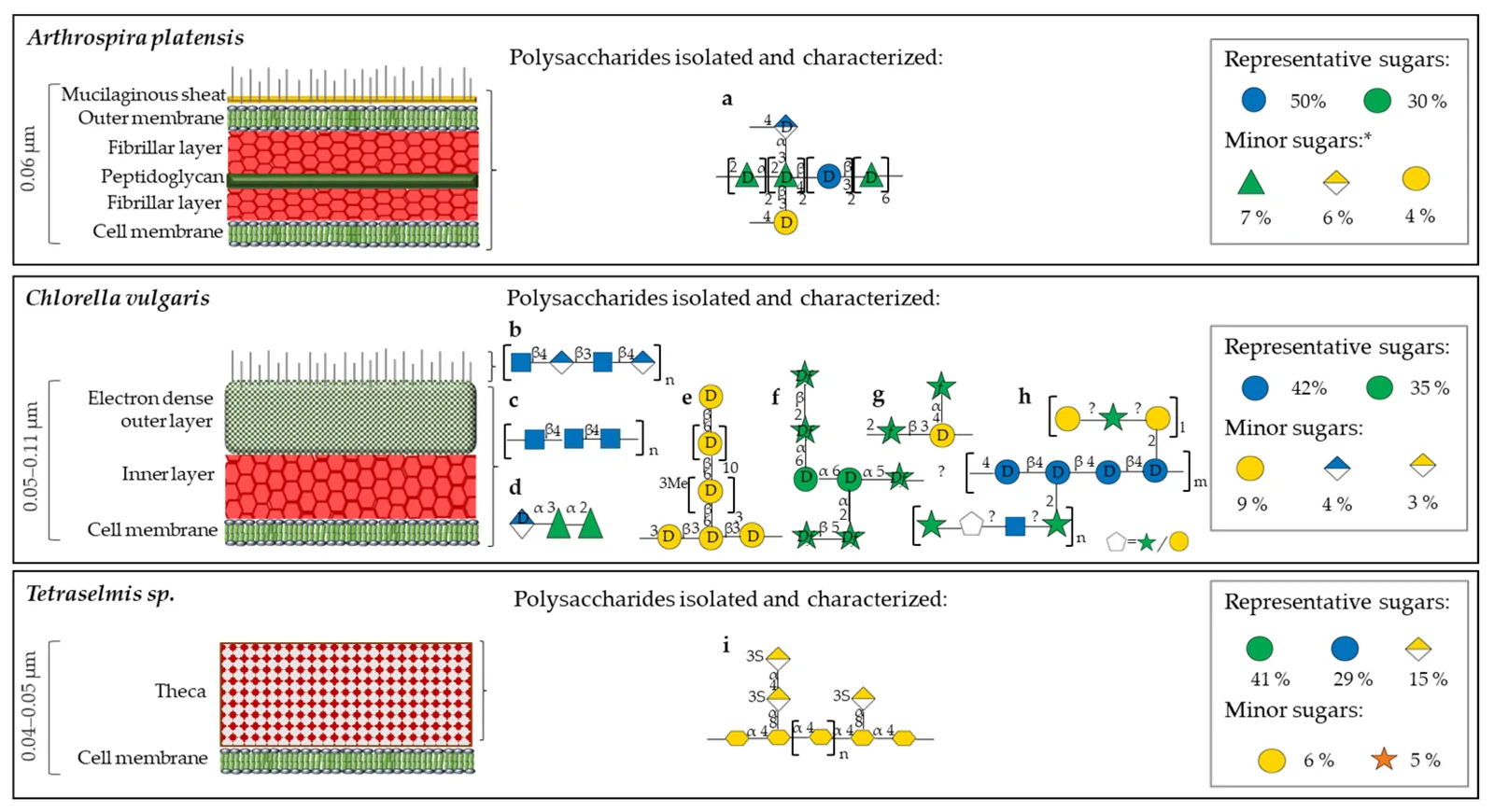
Representative structures of cell-wall polysaccharides reported in *Arthrospira platensis*, *Chlorella vulgaris*, and *Tetraselmis* spp.: (**a**) SP90–1, an acidic heteropolysaccharide from *Arthrospira platensis*; (**b**) hyaluronan; (**c**) chitin-like poly-β-(1 → 4)-N-acetylglucosamine; (**d**) glucuronorhamnan; (**e**) β-D-galactan; (**f**) arabinomannan; (**g**) arabinogalactan; (**h**) arabinogalacto-N-acetylglucosamineglucuronan, and (**i**) an acidic polysaccharide from the *Tetraselmis* theca containing 3-deoxy-D-manno-oct-2-ulosonic acid and D-galacturonic acid. Glycan symbols were drawn according to the Symbol Nomenclature for Glycans (SNFG) guidelines (NCBI, 2024). The representative and minor monosaccharide compositions (%) of cell wall polysaccharides (CWPS), based on data from Bernaerts et al. [46]. * Note that the monosaccharide composition (%) shown in the “Representative sugars” and “Minor sugars” keys refers to the overall cell wall composition, which may differ from the specific monosaccharide arrangement shown in the isolated polysaccharide structures (**a**–**i**).

Cyanobacteria, such as Spirulina (*Arthrospira platensis*), possess a cell wall structurally analogous to that of Gram-negative bacteria, organized into four distinct layers: an inner fibrillar layer adjacent to the plasma membrane, a peptidoglycan layer conferring structural rigidity, a second fibrillar layer, and an outer layer covered by an acidic mucilaginous sheath, together reaching a total wall thickness of approximately 0.06 µm [44] (Figure 2). This thin, multilayered yet flexible architecture facilitates direct extraction of intracellular components without extensive mechanical or enzymatic pre-treatment [45].

Cell wall polysaccharides (CWPS) account for approximately 9.7 ± 0.7% of *Arthrospira platensis* dry biomass, a value statistically similar to the 9.3 ± 0.7% observed in *Chlorella vulgaris*, but markedly lower than the 17.0 ± 1.0% found in *Tetraselmis chuii* [46]. The CWPS of *Arthrospira platensis* are mainly composed of glucose (49.8 ± 5.8%) and mannose (29.8 ± 3.7%), with minor amounts of galacturonic acid (5.6 ± 2.9%), galactose (3.8 ± 0.3%), and glucuronic acid (2.2 ± 0.3%) (Figure 2) [46]. More recently, Cai et al. [47] reported a novel acidic heteropolysaccharide from *Arthrospira platensis* (SP90–1), whose backbone is composed of rhamnose and glucose residues and bears galactose- and glucuronic acid-containing side chains (Figure 2i).

The cell wall of *Chlorella vulgaris* exhibits a more complex, eukaryotic organization than the peptidoglycan-based wall of Spirulina. *Chlorella vulgaris* (strain 13-1) was classified as having low resistance because it lacks an algaenan layer [48]. Consistent with this classification, TEM analyses revealed a relatively thin cell wall, which increased only modestly in thickness during growth, from approximately 0.05 to 0.11 μm. According to [31], true *Chlorella* strains may progressively develop a three-layered wall structure as the cells mature. The cell surface may also contain occasional hair-like microfibrils, which have been associated with hyaluronan-like polysaccharides composed of glucuronic acid and N-acetylglucosamine residues (Figure 2b) [49]. According to Baudelet et al. [31], the rigid glucosamine-based fraction typically accounts for 60–66% of the cell-wall dry mass, remaining constant throughout the growth phase, while the more soluble hemicellulosic matrix (22–25%) increases gradually as the culture matures. Gerken et al. [11] further reported that, within this rigid fraction, N-acetylglucosamine occurs predominantly as a chitin-like glycan, poly-β-1,4-D-N-acetylglucosamine (chitin) (Figure 2c). This is consistent with the biomass-level monosaccharide profile of *Chlorella vulgaris* reported by Bernaerts et al. [46], in which glucose (41.5 ± 1.8%) and mannose (34.8 ± 7.6%) were the dominant sugars, alongside galactose (8.6 ± 0.2%), glucosamine (2.9 ± 0.1%), glucuronic acid (4.3 ± 0.1%), and galacturonic acid (3.3 ± 0.2%) (Figure 2).

Compositionally, according to Takeda [50] and Ferreira et al. [51], *Chlorella vulgaris* belongs to the glucosamine-type *Chlorella* group. This chitin-like character is further corroborated by its enzymatic susceptibility: *Chlorella vulgaris* is particularly sensitive to chitinase and lysozyme, with lysozyme shown by TEM to selectively strip away the outer hair-like microfibrils of the cell wall [11]. In these chitin-type species, the flexible sugar matrix is dominated by the neutral sugar residues rhamnose and galactose and includes glucuronorhamnan, a β-(1 → 3)/β-(1 → 6)-linked galactan containing 3-*O*-methyl-D-galactose, arabinogalactan, arabinomannan, and arabinogalactan-N-acetylglucosamineglucuronan. The acidic glucuronorhamnan was isolated from *Chlorella vulgaris* (K-22) (Figure 2d) [52]. Structural analysis of the hydrolysis products identified the trisaccharide α-D-glucopyranuronosyl(1 → 3)-α-L-rhamnopyranosyl-(1 → 2)-α-L-rhamnopyranose, revealing a characteristic uronic acid–rhamnose–rhamnose sequence. A related aldobiuronic acid, 3-O-α-D-glucopyranuronosyl-L-rhamnopyranose, was previously reported from the same acidic polysaccharide [52]. This finding, together with the presence of a β-(1 → 3)/β-(1 → 6)-linked galactan containing 3-*O*-methyl-D-galactose, highlights the structural diversity of the *Chlorella vulgaris* K-22 cell wall polysaccharides (Figure 2e) [53].

The arabinomannan component, isolated by subsequent NaOH extractions, consists of terminal, 2-*O*-, and 5-*O*-linked arabinofuranosyl residues together with 2,6-*O*-linked mannopyranosyl units, and shows notable structural similarity to the (lipo)arabinomannans found in the mycobacterial cell wall (Figure 2f) [54]. Arabinogalactan (Figure 2g) was isolated from a *Chlorella* extract and characterized as a unique, highly branched polysaccharide consisting of a β-D-galactan backbone substituted with α-L-arabinofuranosyl residues [55]. Sugimoto et al. [56] also described a glucuronic acid-rich polysaccharide bearing side chains containing arabinose, galactose, and N-acetylglucosamine residues, likely attached at C-2 (Figure 2h).

*Tetraselmis* sp. is enclosed not by a conventional polysaccharide wall but by a theca, formed by the extracellular fusion of Golgi-secreted organic scales [57]. Consistent with this fused-scale structure, no released extracellular polysaccharides have been detected [46]. Compositionally, the theca contains up to 80% acidic polysaccharides, including 3-deoxy-manno-2-octulosonic acid, 3- deoxy-5-O-methyl-manno-2-octulosonic acid, and 3-deoxy-lyxo-2-heptulosaric acid, which confer a strongly anionic character to the *Tetraselmis* surface (Figure 2i) [46,57]. Additional minor constituents include D-galacturonic acid (21%), D-galactose (7%), D-gulose (4%), and L-arabinose (1%), while the theca’s protein content is comparatively low, at approximately 3.8% [57]. Other constituents comprise calcium, which accounts for approximately 4% of the dry weight, sulfate (6%), and protein (4%, estimated from the total nitrogen content) [58]. Consistent with this acidic, uronic acid-rich composition, Bernaerts et al. [46] reported that the biomass-level CWPS profile of *Tetraselmis chuii* was composed of mannose (41.3 ± 3.4%), glucose (28.9 ± 4.1%), galacturonic acid (15.1 ± 1.3%), galactose (5.7 ± 0.9%), and xylose (5.3 ± 0.7). Other constituents comprise calcium (about 4% of dry weight), and sulfate (6%) [58].

Discrepancies in the reported cell wall structure of *Arthrospira*, *Chlorella*, and *Tetraselmis* species have been repeatedly described in the literature. In the case of *Chlorella*, these discrepancies can be partly attributed to taxonomic revisions that redefined the genus and transferred previously assigned species to other genera [46], while more broadly, such discrepancies may also reflect physiological differences arising from changes in cultivation conditions or cell states [59].

### 2.3. Protein Content in Microalgae

#### 2.3.1. Mechanisms of Protein Synthesis in Microalgae

Protein production in microalgae is driven primarily by photosynthesis. This process involves two multiprotein complexes, photosystem I and photosystem II, which are located in the chloroplasts of microalgae or within the thylakoid membranes of cyanobacteria [33]. During photosynthesis, microalgae utilize sunlight to convert carbon dioxide (CO_2_) and water into oxygen and organic carbon compounds. These photosynthetic products provide carbon skeletons and energy that, together with assimilated nitrogen, support amino acid and protein biosynthesis [33]. The energy from sunlight is initially captured and stored as ATP and NADPH during the light-dependent reactions. This energy then fuels carbohydrate synthesis through the Calvin cycle.

A significant proportion of microalgal proteins is associated with the chloroplast, the primary photosynthetic organelle of the cell, while water-soluble proteins are also present in the cytoplasm [36]. Cyanobacteria contain carboxysomes, specialized microcompartments that facilitate CO_2_ fixation through ribulose-1,5-bisphosphate carboxylase/oxygenase (RuBisCO), the key enzyme of the Calvin cycle. Moreover, cyanobacteria possess phycobilisomes, which are composed of phycobiliproteins and play a crucial role in light capture during photosynthesis. Similar structures are also found in certain algal species, although they are not universally present across all taxa [13].

Protein synthesis in microalgae is significantly influenced by environmental and cultivation conditions. Factors such as light intensity, nutrient availability (notably nitrogen and phosphorus), temperature, and CO_2_ concentration affect both the rate of photosynthesis and overall protein content [4]. Moreover, microalgae can synthesize specific proteins in response to environmental stressors, such as ultraviolet radiation, salinity fluctuations, and temperature variations [60]. Consequently, reported protein levels reflect both baseline metabolism and adaptive responses, meaning that protein content is a dynamic trait rather than a fixed species characteristic.

The protein content of microalgae (proportion of protein in whole biomass) varies significantly both inter- and intra-species. This variability arises from genotype, cultivation strategy (medium composition, light and temperature regimes, cultivation mode), harvest timing, and post-harvest handling, as well as from differences in analytical methods used for protein quantification [61]. Therefore, direct comparisons between studies require careful standardization of these variables.

For clarity, protein content is defined as the proportion of protein in the whole biomass or in an extract, generally expressed as a percentage of dry weight (dw).

#### 2.3.2. Protein Content and Amino Acid Profile in Microalgae

**Figure 3:**
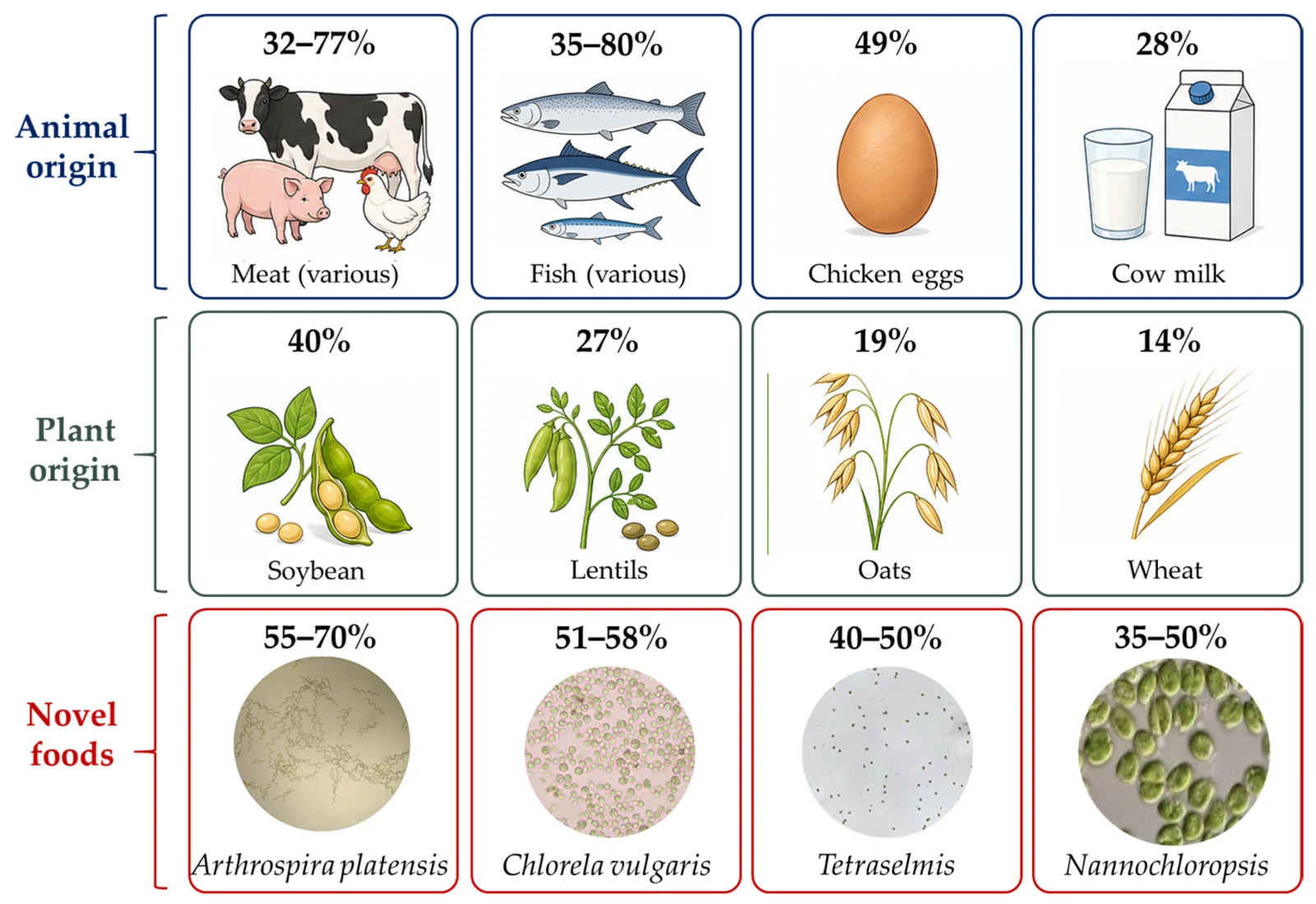
Comparative analysis of protein content in novel foods, such as microalgae (data from [62]), and traditional animal- and plant-based foods (data from [63,64,65,66,67,68]), with all values expressed on a dry weight (dw) basis.

Microalgae are notably rich protein sources, as illustrated in Figure 3, which compares their protein content, expressed on a dry weight (dw) basis, with that of traditional animal- and plant-based protein sources. Their growing incorporation into food products highlights their potential as sustainable and versatile protein ingredients.

Spirulina typically contains 55–70% protein on a dw basis, whereas *Chlorella vulgaris Tetraselmis* spp., and *Nannochloropsis* spp. contain approximately 51–58%, 40–50%, and 35–50% protein, respectively. [62]. These values are comparable to, or in some cases exceed, those reported for conventional plant-based sources, such as soybeans (40% dw), and animal-derived sources, such as chicken meat (77% dw) and beef, pork, and lamb (32–48% dw) [63,64,65,66,67,68].

In addition to total protein, Spirulina biomass contains water-soluble phycobiliproteins, which are macromolecular complexes composed of covalently attached open-chain tetrapyrroles known as phycobilins [4]. The main phycobiliproteins include C-phycocyanin and allophycocyanin, which are of interest as natural colourants and functional ingredients in food, pharmaceutical, and biotechnology industries [8].

Table 1 presents the amino acid profiles of proteins derived from Spirulina, *Chlorella vulgaris*, and *Tetraselmis chuii*. These values refer to whole-biomass amino acid profiles, rather than isolated protein fractions. Differences between studies may further arise from strain identity, culture conditions, post-harvest handling, hydrolysis procedures, and analytical methods [45]. The distinction between whole biomass and isolated protein fractions is particularly relevant for *Spirulina*. Costa et al. [14] showed that extraction and precipitation conditions affected the amino acid composition, protein purity, phycobiliprotein content, and structural properties of the resulting extract. Therefore, amino acid values reported for whole biomass should not be directly extrapolated to purified protein fractions intended for specific food or oenological applications.

In Spirulina, studies by Bashir et al. [69] and Teuling et al. [10] identified leucine as the most abundant essential amino acid (6.2–9.3 g/100 g), while histidine was the least abundant (1.1–1.8 g/100 g). Among the non-essential amino acids, glutamic acid + glutamine were the most prevalent (8.5–14.6 g/100 g), whereas cysteine, a conditionally indispensable amino acid that can be synthesised from methionine, was present at comparatively low concentrations (0.6–0.8 g/100 g).

A similar pattern was observed in *Chlorella vulgaris*, in which leucine was the most abundant essential amino acid (1.9–4.5 g/100 g), whereas histidine occurred at the lowest concentration (0.6–1.1 g/100 g protein). Among the non-essential amino acids, alanine (2.4–4.4 g/100 g) and glutamic acid + glutamine (3.5–6.3 g/100 g) were the most prevalent, whereas cysteine (0.3–0.7 g/100 g) and tyrosine (0.7–2.2 g/100 g), conditionally indispensable amino acids synthesised from methionine and phenylalanine, respectively, occurred at comparatively low concentrations [37,70].

Similarly, in *Tetraselmis chuii*, leucine was the most abundant essential amino acid (2.3–4.9 g/100 g), whereas histidine occurred at the lowest concentration (0.6–0.8 g/100 g protein). Among the non-essential and conditionally indispensable amino acids, amino acids, glutamic acid + glutamine (4.8–8.6 g/100 g) and aspartic acid + asparagine (5.2–6.5 g/100 g) were the most prevalent, whereas cysteine, a conditionally indispensable amino acid synthesised from methionine, occurred at comparatively low concentrations (0.5–1.2 g/100 g protein) [37,70]. Differences in absolute and relative amino-acid levels among Spirulina, *Chlorella vulgaris*, and *Tetraselmis chuii* may reflect taxonomic differences in protein composition.

Comparative analysis indicates that Spirulina contains higher or comparable concentrations of certain essential amino acids compared with commonly consumed protein sources. For instance, methionine (1.7–2.5 g/100 g), which is often present at relatively low levels in plant-based proteins, is present at relatively high concentrations in Spirulina. Lysine (3.4–4.9 g/100 g), which is typically limited in cereals and other plant-based proteins, is found in moderate amounts, further enhancing the nutritional value of Spirulina biomass.

Protein quality is typically assessed using the amino acid score (AAS), which evaluates the balance of essential amino acids in a protein relative to a reference amino acid pattern established by the FAO/WHO/UNU [72]. An AAS value ≥ 1 indicates that all essential amino acids meet or exceed dietary requirements, with higher values generally reflecting greater protein bioavailability and utilization efficiency [73]. Notably, microalgal proteins exhibit the following AAS values: Spirulina, 1.37 [74]; *Chlorella vulgaris*, 1.10 [73]; and *Tetraselmis chuii,* 1.00–1.07 [73]. However, AAS does not fully capture biological value because it does not account for differences in digestibility [73]. The protein-digestibility-corrected amino acid score (PDCAAS) and the digestible indispensable amino acid score (DIAAS) provide more comprehensive assessments by incorporating protein digestibility. PDCAAS values have been reported for some microalgae; for example, mechanical cell disruption increased the PDCAAS of *Chlorella vulgaris* from 0.63 to 0.77 [73]. However, DIAAS data remains limited and is unavailable for most microalgal species. In addition to nutritional quality, amino acid composition can also influence technological performance. In a purified *Spirulina* protein extract used as a model wine fining agent, descriptors related to charge, hydrophobicity, flexibility, and hydrogen-bonding capacity were associated with proanthocyanidin removal. These findings illustrate that protein composition may affect both nutritional value and protein–polyphenol interactions [14].

## 3. Protein Extraction

Protein recovery is defined as the percentage of the protein initially present in the biomass that is recovered after a given process step. The presence of proteins and other intracellular compounds within microalgal cells necessitates cell disruption to facilitate their release and subsequent recovery [75]. This process can be achieved using mechanical or non-mechanical methods, each with distinct advantages and limitations. However, extraction performance is not determined solely by the disruption method: it also depends on cell-wall architecture, species, biomass state, and operating conditions, which together explain much of the variability reported across studies. As established in Section 2.2, the structural differences between Spirulina, *Chlorella vulgaris*, and *Tetraselmis chuii* directly determine which extraction strategy is most appropriate for each species, and this relationship is reflected throughout the comparisons presented below. Recent work on *Spirulina* highlights the importance of jointly optimizing protein content and recovery yield. Costa et al. [14] compared bead milling and ultrasound-assisted extraction and identified bead milling as the more practical option, as it provided comparable performance while requiring less complex equipment and offering greater scale-up potential.

Mechanical techniques are among the most widely used approaches for breaking down the tough cell walls of microalgae, ensuring efficient protein extraction. Common methods include high-pressure homogenization [76], ultrasonication using either bath or probe sonicators [77], and bead milling [9,75]. These methods are generally effective because they physically overcome rigid cell walls, but they also differ in terms of energy demand, scalability, and the risk of protein damage. Therefore, the most appropriate method depends on the desired outcome, whether maximum protein recovery, preservation of protein integrity, or industrial feasibility.

Non-mechanical methods, such as enzymatic lysis and chemical treatments [11], provide a gentler alternative for cell disruption [15]. These approaches can help preserve the structural integrity and functionality of sensitive proteins, thereby reducing degradation during the extraction process. Their main advantage are selectivity and milder processing conditions; however, they are usually slower, more expensive, and more sensitive to enzyme specificity or pH control, which can limit scalability.

Combining different disruption techniques can further enhance the extraction process by maximizing cell wall disruption and improving protein recovery [15]. This explains why combined strategies often outperform single-step methods: physical disruption opens the cells, while enzymatic or chemical steps improve solubilization and release proteins that would otherwise remain inaccessible.

Table 2 presents an overview of the most efficient, straightforward, and cost-effective cell disruption methods currently used for protein extraction from microalgae. Additionally, Table 3 summarizes the extraction methods applicable to different microalgal species.

### 3.1. Physical Methods

#### 3.1.1. High-Pressure Homogenization (HPH)

HPH is a cell disruption technique that utilizes high pressure to accelerate a fluid jet, which then impacts a stationary valve surface [76]. This process generates intense hydrodynamic cavitation due to the shear stress created by the sudden pressure drop across the valve [15]. The energy released within this confined environment results in highly effective cell disruption and the subsequent release of intracellular components [15,45].

HPH is one of the most effective physical methods for microalgal cell disruption, providing high efficiency and short processing times [16]. However, it requires high operating pressures, careful control of pressure and temperature to minimize heat- or shear-induced protein damage, and costly, energy-intensive equipment, which can limit its scale-up and increase operational costs [76]. Safi et al. [45] compared high-pressure homogenization (HPH) with chemical treatments, ultrasonication, and manual grinding for protein extraction from Spirulina and *Chlorella vulgaris*. HPH (*p* = 270 MPa, np = 2) was identified as the most effective method, achieving protein recoveries of 78.0 ± 2.8% and 52.8 ± 0.6% of the total protein content of Spirulina and *Chlorella vulgaris*, respectively.

Similarly, Carullo et al. [78] demonstrated that high-pressure homogenization (*p* = 150 MPa, np = 5) completely disrupted *Chlorella vulgaris* cells, leading to the rapid release of intracellular components, including approximately 33.0% of proteins (% dw biomass).

Magpusao et al. [18] investigated the optimal pressure levels for HPH treatment of *Tetraselmis* species, observing substantial cell disintegration at 30 MPa and complete disruption at 90 MPa.

Similarly, Kulkarni and Nikolov [79] reported a protein recovery of 75.6 ± 3.8% from *Chlorella vulgaris* using high-pressure homogenization (HPH; *p* = 103 MPa, np = 5). This value was used as a reference for comparison with ultrasonication and bead milling. Ultrasonication, applied for 30 min, and bead milling, applied for 15 min achieved protein recoveries of 76.6 ± 1.0% and 75.5 ± 0.5%, respectively. The three methods showed comparable protein recovery, however, HPH was identified as the most effective method in terms of productivity and scalability because of its shorter total processing time of approximately 9 min (6 min plus 3 min).

Protein solubilization yield refers to the percentage of protein initially present in biomass that is transferred to the soluble fraction during the extraction. Ursu et al. [96] reported the highest protein solubilization yield using high-pressure cell disruption (*p* = 270 MPa, *n* = 2) combined with alkaline extraction at pH 12, yielding 52 ± 3% (*w*/*w*) protein solubilization. The crude protein content of the initial biomass was estimated at 53% (*w*/*w*).

More recently, Rida et al. [80] demonstrated that HPH (*p* = 30 MPa, np = 2) was superior for extracting proteins and pigments from *Tetraselmis suecica*, achieving 61.9 ± 3.9% protein extraction, compared with ultrasound probe methods, which extracted 44.8 ± 1.3% of the proteins.

#### 3.1.2. Ultrasonic-Assisted Extraction (UAE)

UAE utilizes high-energy ultrasonic waves to break down microalgal cell walls, facilitating the release of intracellular compounds through the formation and collapse of cavitation bubbles [17]. The technique can be performed using either an ultrasonic bath system or a probe system. In ultrasonic bath systems, acoustic waves are transmitted indirectly within the sample, whereas in ultrasonic probe systems, the waves are directly transmitted into the sample via a probe inserted into the liquid phase. Its main advantages are rapid processing and reduced solvent use, but high local heating, energy consumption, and possible protein degradation can limit its performance if the operating conditions are not carefully controlled.

Yucetepe et al. [83] optimized ultrasound-assisted extraction conditions for Spirulina, demonstrating that the most effective parameters were 45 °C, pH 7.46, and 120 min of extraction time. Under these conditions, the protein content of the starting material was 65.6 ± 0.1% (dw), and the extraction recovery ranged from 20.03% to 29.96%. These results highlight the importance of optimizing sonication parameters to maximize protein recovery while minimizing degradation. The relatively modest recovery indicates that ultrasound alone may not fully overcome matrix resistance, even when the process is optimized.

Similarly, Lupatini et al. [81] evaluated the co-extraction of proteins from Spirulina using an ultrasound probe combined with mechanical agitation under alkaline conditions. The starting Spirulina biomass contained 50.75% (dw) protein, and under optimized conditions (30 g/L biomass, pH 9.0, 35 min sonication, followed by 50 min mechanical agitation), they achieved a protein recovery of 75.76%, demonstrating the effectiveness of combining ultrasonic and mechanical forces to enhance extraction efficiency.

Safi et al. [45] successfully applied probe ultrasonication (20 kHz, 13 mm) for 30 min, using 5 s ultrasonication pulses followed by 15 s rest intervals, to extract proteins from Spirulina and *Chlorella vulgaris*. The total protein content of crude Spirulina and *Chlorella vulgaris* biomass, determined using the Lowry method, were 53.5 ± 1.10% (dw) and 49.6 ± 1.04% (dw), respectively. After ultrasonication, the fraction of soluble protein recovered was 47.1 ± 0.9% (dw) for Spirulina and 18.1 ± 0.0% (dw) for *Chlorella vulgaris*. However, the efficiency for *Chlorella vulgaris* was notably lower, suggesting that ultrasonication may not be the optimal method for this species due to its more rigid cell wall structure. This species difference highlights a key limitation of UAE: although it can be highly effective for more fragile matrices, it may be less effective for cell walls that resist cavitation-induced rupture.

Zhang et al. [77] evaluated protein extraction from *Nannochloropsis* sp., *P. tricornutum*, and *P. kessleri* using pulsed electric fields (40 kV–10 kA), high voltage electric discharges, and ultrasound (24 kHz frequency). For all species tested, ultrasonic assisted extraction (UAE) achieved higher protein recovery than high-voltage electric discharges and pulsed electric fields. These findings suggest that UAE offers a favorable balance between protein recovery and processing time, provided that thermal effects are carefully controlled. The application of sonication during extraction results in increased temperature and pressure [3], leading rapid processing times and high-efficiency cell wall disruption. However, protein structures may be compromised under such conditions. Therefore, optimization of extraction parameters, such as time and temperature, is essential to prevent heat-induced protein degradation [86].

Obeid et al. [98] optimized low-frequency ultrasound-assisted extraction for protein recovery from *Chlorella vulgaris*. Using an ultrasonic reactor operated at 12 kHz and 100–300 W, at 30 °C for 1 h under continuous stirring, they reported a protein content of 58.0 ± 1% (dw) in the *Chlorella vulgaris* biomass. Protein recovery increased with ultrasonic power, achieving 64.3% and 94.1% of the total protein at 100 W and 300 W, respectively. These findings highlight the effectiveness of low-frequency ultrasound as a green protein extraction technology. However, increasing power may also increase energy consumption and the risk of unwanted co-extraction or structural damage if operating conditions exceed the optimum.

Similarly, Hildebrand et al. [99] used an ultrasound probe (35–130 kHz) combined with enzymatic treatment (protease and lysozyme) and sequential solvent extraction (water at pH 7, HCl 0.4 M, NaOH 0.4 M). The highest protein recoveries were obtained with ultrasound-assisted enzymatic extraction, although alkaline ultrasound-assisted extraction produced comparable results. To further improve protein recovery and purity, additional modifications can be employed, including the incorporation of salts (e.g., NaCl), adjustment of buffer pH, or enzymatic digestion (e.g., using lysozyme) [82]. In line with this, Milia et al. [97] reported that the use of phosphate-buffered saline followed by ultrasound-assisted extraction not only facilitated the solubilization of polar lipids, such as glycerophospholipids, and the disruption of protein–lipid complexes, but also maintained high protein content—62.66% (dw) in Spirulina biomass and 52.23 ± 0.5% (dw) after extraction.

While ultrasonication is widely used in laboratory settings for small-scale cell disruption due to its precision and efficiency [100], scaling up the process remains a challenge. Large-scale ultrasonic equipment is expensive, and complete protein extraction is often difficult to achieve [82]. Similarly, Khalid et al. [101] emphasized that ultrasound-assisted extraction is among the most efficient innovative techniques for recovering proteins from Spirulina, as it can provide higher protein recovery and reduce protein denaturation when parameters such as power, frequency, and treatment time are carefully optimized. However, issues related to scalability and energy efficiency still limit its industrial application. Thus, ultrasound is attractive for rapid protein extraction, but its industrial relevance depends on whether the benefits in protein recovery justify the associated equipment and energy burden. In comparison with HPH, UAE is often more flexible in laboratory-scale use but less straightforward to scale industrially.

#### 3.1.3. Bead Milling

Bead milling is a widely used industrial cell disruption technique [17] that can be adaptable to both small- and large-scale protein extraction from microalgae [3]. This non-thermal and relatively environmentally friendly method relies on the mechanical agitation of beads to disrupt algal cells [15], thereby facilitating cell disruption and the release of water-soluble proteins [45]. Its main advantages include scalability, non-thermal processing, and established industrial applicability, while its main limitation is that protein recovery can be lower than that achieved with high-pressure methods unless it is combined with a subsequent solubilization or enzymatic treatment. A recent study optimized bead milling for protein extraction from *Arthrospira platensis*, using pH and NaCl concentration as the main process variables. The selected condition, pH 7.0 and 1 M NaCl, followed by ethanol precipitation, yielded an extract containing approximately 63% protein, with a protein recovery of approximately 25% [14].

Protein yield refers to the mass of protein recovered per unit of biomass or process input and is commonly expressed as g protein/100 g biomass. Jaeschke et al. [87] compared pulsed electric fields (40 kV·cm^−1^) with bead milling (4 cycles of 25 s at 30 Hz) for protein extraction from Spirulina. Bead milling produced a protein yield of 46.1 ± 4.4 g protein/100 g biomass, whereas pulsed electric field extraction achieved 46.9 ± 3.5 g protein/100 g biomass, indicating that both techniques resulted in comparable protein yields under the conditions investigated. Ng et al. [88] optimized protein extraction from *Chlorella vulgaris* by combining bead milling (26 Hz, 1 h) with alkaline solubilization using NaOH at a biomass to NaOH molar ratio of 200–250 g/M, 37 °C, and 1 h. The solubilized protein was subsequently recovered by acid-induced precipitation, achieved by lowering the extract pH to 1.0 with HCl. The protein extraction recovery, calculated as the amount of solubilized protein in the supernatant relative to the total protein initially present in the biomass and measured using the Bradford assay, reached a maximum of 47.3% at a biomass loading of 100 g/L. In another study, Schwenzfeier et al. [89] extracted proteins from *Tetraselmis* sp. using a multi-step process involving bead milling, centrifugation, ion exchange chromatography with a Streamline DEAE absorbent, and final decolorization by precipitation at pH 3.5. The initial protein content of the *Tetraselmis* sp. biomass, determined by the Dumas method, was 36% (*w*/*w*). Following bead milling and centrifugation, 24.5% (*w*/*w*) of the total protein was recovered, yielding a total water-soluble protein content of 21%. This illustrates an important trade-off: multi-step processing can improve purity and decolorization but often at the expense of recovery and process simplicity. Thus, although bead milling is attractive for scale-up, however high purity often requires additional downstream steps.

Similarly, Suarez Garcia et al. [9] extracted protein from *Tetraselmis suecica* using a single bead milling step. The total protein content of the biomass, measured using the Lowry method, was 54.0 ± 4.0% (dw), while the extracted protein fraction contained 50.4% (dw) protein. Differences in protein content across studies were attributed to variations in algal culture conditions [9] and protein quantification methods. These differences also reflect the physiological state of the biomass and the extent of cell-wall resistance at harvest.

Alavijeh et al. [75] investigated the combination of bead milling (2039 rpm and 25 °C) and enzymatic hydrolysis (2% [*w*/*v*] phospholipase, 37 °C, 24 h) to extract protein from *Chlorella vulgaris*. Bead milling alone resulted in a protein recovery of 40%, whereas the addition of enzymatic hydrolysis led to a substantial increase, achieving a protein recovery of 68% from *Chlorella vulgaris*.

Although bead milling resulted in lower protein recovery than high-pressure homogenization [45], its combination with enzymatic or chemical treatments has been shown to significantly enhance overall protein recovery [17].

Overall, HPH offers short processing times and high disruption efficiency, whereas UAE provides greater operational flexibility but may be constrained by energy demand and scale-up limitations. Bead milling is mechanically simpler and highly scalable, although additional solubilization steps may be required to achieve comparable protein recovery. Therefore, process selection should consider not only protein recovery but also equipment cost, energy demand, operational flexibility, protein functionality, and overall industrial feasibility.

Table 2 summarizes the main cell disruption methods in terms of protein recovery, energy consumption, cost, scalability, preservation of protein functionality, and main limitations. HPH and combined disruption-extraction methods generally provide the highest protein recovery, but they require high energy input, and combined methods involve greater process complexity and cost. UAE can achieve comparable recovery under optimized conditions, although its high energy demand and moderate scalability may limit industrial application. Bead milling offers the highest scalability, lower energy consumption, and good preservation of protein functionality, but often requires an additional solubilization or enzymatic step to improve recovery. Enzymatic lysis is particularly suitable when mild processing conditions and functionality preservation are priorities, whereas chemical treatment may offer a simpler and potentially lower-cost alternative but can affect protein integrity and raise environmental concerns. Therefore, method selection should consider the microalgal species, target protein recovery, processing scale, cost, energy demand, protein functionality, and intended application of the final protein extract.

### 3.2. Enzymatic and Chemical Treatments for Protein Extraction

Relying on a single protein extraction method may result in incomplete cell disruption and low protein recovery efficiency [93]. Researchers have found that optimizing extraction conditions, particularly when combining physical disruption with enzymatic, or chemical treatments, can significantly enhance protein release [18,93]. Since optimal extraction conditions vary among microalgal species, tailoring extraction methods to specific species characteristics is essential for improving process efficiency.

Enzymatic treatments target specific components of the cell wall structures to facilitate selective degradation and digestion, thereby aiding in th protein separation from polysaccharides [15,19]. In some cases, the use of multiple enzymes is necessary to achieve effective cell disruption [11]. The specificity of enzymes plays a crucial role, as different enzyme preparations target different cleavage sites, thereby influencing the protein hydrolysis pattern based on the composition of the microalgal cell wall [86]. For example, cellulases, such as *Trichoderma reesei* cellulase, effectively degrade cellulose within the cell wall [102]. Lysozyme, a hydrolytic enzyme, cleaves β-(1,4)—glycosidic bonds in peptidoglycan, a major component of bacterial cell walls [90]. The main advantage of enzymatic extraction is its selectivity and the use of mild processing conditions, whereas its disadvantages are long reaction times, relatively high cost, and the need to match the enzyme type to the specific cell wall composition.

Enzyme-assisted extraction is considered a relatively gentle process compared with other methods, such as alkaline or salt-based extraction. It is particularly valued for producing protein fractions with high purity while minimizing protein denaturation and degradation. However, it is often associated with higher costs and longer processing time [77].

Martins et al. [92], examined the effectiveness of various physical (aqueous extraction, ultrasonication, and cell homogenization), chemical (alkaline pre-treatment with 2M NaOH at pH 12), and enzymatic (cellulase mixture and alcalase) techniques for extracting proteins from *Chlorella vulgaris*. The study found that alcalase was more effective than cellulase, recovering approximately 80% of the initial protein from *Chlorella vulgaris* biomass containing 44.7 g protein/100 g dry weight, primarily in the non-acid-precipitable fraction. These findings highlight the potential of alcalase as an alternative to physical cell-disruption methods, although it should be noted that alcalase is a non-specific protease and may therefore hydrolyze proteins in addition to disrupting the cell matrix.

Similarly, Yucetepe [91] employed a combination of osmotic shock, carbohydrase enzymes, and ultrasonic waves to extract proteins from *Chlorella vulgaris*. Under optimized conditions-an enzyme/substrate ratio of 0.9, an ultrasound probe treatment time of 120 s, and an extraction time of 2.0 h the highest protein content achieved was 26.3 g protein/100 g dw.

In addition, Schreiber et al. [19] reported that nylon membrane grafted with lipase (5 g/L) and trypsin (15 g/L) enhanced protein recovery from *Tetraselmis chuii* extracts, achieving protein recoveries of 24% at a biomass concentration of 10 g/L and 40% at 1 g/L. Furthermore, Poidevin et al. [20] demonstrated that coupling enzymatic hydrolysis with microfiltration could substantially improve protein recovery from *Tetraselmis chuii*. Using a lipase–pectinase enzyme mixture, up to 73% of proteins in the supernatant were recovered, compared with only 25% using enzyme-free filtration. Alternative enzyme combinations (trypsin–lipase or trypsin–pectinase) achieved approximately 41% recovery but resulted in higher membrane permeability. These results underline a key trade-off: more aggressive or better-matched enzyme systems can improve recovery, but they can also increase process complexity and influence membrane fouling/permeability.

Al-Zuhair et al. [103] investigated enzymatic pre-treatment using cellulase and lysozyme prior to protein extraction from various microalgal strains, including *Chlorella* sp. and *Tetraselmis* sp., followed by ultrasonication and high-pressure water extraction. Enzymatic pre-treatment increased protein yields for all tested strains to approximately 0.7 mg protein per mg of dry cell weight, and lysozyme treatment also enhanced pigment extraction, demonstrating its potential for improving the recovery of multiple intracellular compounds.

Chemical treatments, including the use of salts or organic solvents, can facilitate cell lysis through relatively simple and effective methods, often involving pH adjustments, such as alkaline protein dissolution and acid-mediated separation [86]. However, these methods carry the risk of protein degradation, which can limit protein recovery [93]. The main advantage of chemical treatments are their relative simplicity and speed; their main disadvantage is that harsh conditions may reduce protein integrity and increase downstream purification needs.

Combined extraction approaches that integrate enzymatic or chemical treatments with physical disruption techniques have shown promise for improving protein recovery. However, these methods are often cost-prohibitive due to the high expenses associated with enzymes and reagents [94]. Additional challenges include allergenicity and sustainability concerns, particularly for enzymes like lysozyme, which is frequently derived from animal sources [104]. This limits their application in allergen-free or vegan-friendly product development.

To address these issues, alternative strategies are being explored, such as recycling pH-adjusting agents and ensuring that lysozyme is absent from the final extracts. These approaches aim to reduce processing costs, enhance sustainability, and minimize potential allergenic risks.

The properties of the resulting protein extract (its purity, functionality, and structural integrity) depend directly on these upstream choices. Therefore, the recovery and purification steps discussed in Section 4 (centrifugation, precipitation, dialysis, and ultrafiltration) should be viewed as a continuation of the same extraction process rather than as an independent downstream operation.

## 4. Protein Precipitation and Purification

After disrupting microalgal biomass, centrifugation is commonly used to remove insoluble components, such as cell debris, yielding a supernatant rich in soluble proteins [45]. Protein purity refers to the proportion of protein relative to non-protein constituents, such as polysaccharides, pigments, lipids, and salts; it therefore reflects separation quality rather than protein quantity recovered. This protein-rich fraction can then undergo precipitation to enhance protein recovery. Common precipitation methods include isoelectric precipitation [84,85,105] organic solvent precipitation [106], and salting out techniques [107].

Protein precipitation is typically achieved by adding a suitable precipitating agent to the protein solution, followed by centrifugation to separate the precipitated proteins from the liquid phase [1]. The recovered protein fraction may then undergo further purification steps to improve its purity, quality and functionality. This is particularly important for microalgal protein extracts, which may contain pigments, phenolic compounds, salts, and other low-molecular-weight compounds that can negatively affect their functionality, sensory properties, or suitability for food applications. Further purification can be achieved through membrane-based methods, such as dialysis and ultrafiltration, which are particularly attractive due to their operational simplicity, scalability, and potential cost-effectiveness. Their main advantage is the selective removal of small molecules and other low-molecular-weight contaminants while retaining protein and, under appropriate conditions, preserving their functional properties., but their main drawback is that protein recovery can decrease if proteins are lost during membrane processing, adsorption to the membrane, or membrane fouling, which can reduce overall process efficiency.

### 4.1. Precipitation Methods

#### 4.1.1. Isoelectric Precipitation

The isoelectric precipitation method involves adjusting the pH of a protein solution to the isoelectric point (pI) of a specific protein, at which its net charge becomes zero, leading to reduced solubility and subsequent precipitation. However, this approach may be less effective for the selective separation of complex protein mixtures, as different proteins often have overlapping isoelectric points, which can result in co-precipitation and reduced specificity [1].

Sánchez-Zurano et al. [84] optimized isoelectric precipitation for Spirulina protein, identifying a pH of 3.89 and a precipitation time of 45 min as the optimal conditions. These conditions resulted in an extract with a protein content of 79.1 ± 1.8%.

Similarly, Silva et al. [85] applied ultrasound-assisted extraction using an ultrasonic bath (25 Hz), followed by isoelectric precipitation (pH 3.00), obtaining an extract yield of 43.6 ± 0.93 wt% and a protein content of 66.6 ± 0.3 wt% in the resulting extract.

Özsoy et al. [108] also evaluated alkaline extraction followed by isoelectric precipitation (pH 3.0) for Spirulina, obtaining a protein concentrate with 57.3 ± 0.6% (dw) protein content and a protein recovery of 13.0 ± 1.4%. These results indicate that, despite its relatively low recovery, the method provides an effective means of concentrating proteins.

Additionally, Pereira et al. [105] demonstrated that high-speed homogenization combined with protein solubilization at pH 11 and precipitation at the isoelectric point (pH 4.2) produced a Spirulina protein concentrate with 83.9 ± 1.7 wt % protein and a Spirulina protein isolate with 91.3 ± 1.2 wt % protein. The initial biomass of Spirulina sp. contained 53.8 wt% protein, highlighting the effectiveness of isoelectric precipitation in increasing protein purity.

According to Ursu et al. [96], after protein extraction from *Chlorella vulgaris* using a high-pressure disrupter and solubilization at pH 12, precipitation at pH 4 (the point of minimum solubility for major protein fractions) was performed to recover the proteins. Subsequent solubilization tests at pH 12 and 7 resulted in protein recovery rates of 76 ± 4% (*w*/*w*) and 57 ± 4% (*w*/*w*), respectively.

Overall, isoelectric precipitation is a relatively simple and effective method for concentrating microalgal proteins and increasing protein purity. However, its efficiency is strongly dependent on pH and the composition of the protein mixture. Although high protein purity can be achieved, protein recovery may be limited, and co-precipitation of other biomolecules can occur. Consequently, combining isoelectric precipitation with appropriate upstream extraction and downstream purification steps can improve both recovery and the quality of the final protein preparation.

#### 4.1.2. Organic Solvent Precipitation

The use of organic solvents, such as ethanol, methanol, or acetone, is a common approach for precipitating proteins from aqueous microalgal extracts [106]. These precipitation methods are often performed at low temperatures to minimize protein denaturation and enhance recovery efficiency. Among the solvents commonly employed, ethanol is considered more favorable due to its lower toxicity compared with acetone or methanol. This makes it a more sustainable choice for environmental and operational applications. The advantage of this approach is rapid precipitation and relatively simple implementation, whereas the disadvantage includes the potential for protein denaturation and solvent recovery requirements that can increase process complexity and operating costs. Ethanol precipitation has also been applied to *Spirulina* protein extracts. Following bead milling at pH 7.0 in the presence of 1 M NaCl, precipitation with 75% (*v*/*v*) ethanol yielded a protein isolate containing approximately 63% protein, providing a practical and scalable approach for protein isolation [14].

A study by Grossmann et al. [106] on *Chlorella protothecoides* demonstrated high precipitation efficiency using a 1:1 ethanol: acetone (*v*/*v*) solvent mixture. The resulting extracts contained protein contents of 46.3 ± 0.1 g protein/100 g in the soluble fractions and 67.2 ± 1.0 g protein/100 g in the insoluble fractions. These results highlight the effectiveness of solvent-based precipitation for concentrating microalgal proteins.

In recent years, biodegradable solvents, such as ionic liquids and deep eutectic solvents, have shown potential for efficient protein extraction and precipitation with reduced environmental impact and improved extraction efficiency [109]. However, their industrial application faces several challenges, including high production costs, potential environmental toxicity, solvent recovery requirements, and compatibility issues with certain proteins and microalgal matrices. While ongoing advancements aim to overcome these limitations, further research is needed to improve their scalability, safety, environmental performance, and cost-effectiveness for large-scale microalgal protein processing.

#### 4.1.3. Salting out Method

The salting-out method is a widely used protein separation and purification technique that exploits the ability of salts to reduce protein solubility in aqueous solutions. Among the various salts available, ammonium sulfate is the most commonly employed for protein precipitation [107] due to its cost-effectiveness, simplicity, and scalability. These properties make it suitable for both laboratory and industrial applications.

With proper management, the salt can potentially be recovered and reused, thereby minimizing waste generation and enhancing process sustainability. Additionally, this method selectively precipitates proteins while leaving many non-protein impurities in solution, thereby simplifying downstream purification steps.

Unlike organic solvent precipitation, the salting-out method generally minimizes protein denaturation, helping to preserve the bioactivity of sensitive proteins [1]. However, the efficiency of protein precipitation and the retention of protein functionality depend on several factors, such as salt concentration, pH, protein characteristics, and process temperature. Moreover, residual salt generally needs to be removed or reduced before food applications, which may require additional downstream purification steps.

### 4.2. Membrane-Based Purification Methods: Dialysis and Ultrafiltration

Microalgae are a rich source of phenolic compounds and pigments [110]. For example, the total phenolic content of Spirulina biomass has been reported to be 19.0 mg gallic acid equivalents/g, as measured by the Folin-Ciocalteu method [110]. Due to the presence of these compounds, protein extraction methods often incorporate membrane-based purification techniques, such as dialysis or ultrafiltration, to remove residual salts or solvents after precipitation and to separate proteins from pigments and low-molecular weight compounds including polyphenols.

Dialysis employs semi-permeable membranes with defined molecular weight cut-off values (e.g., 14 kDa) to remove small molecules while retaining proteins. According to Schwenzfeier et al. [89], *Tetraselmis* biomass contains 36% (*w*/*w*) protein. Following bead milling and centrifugation, 21% of the total soluble protein was extracted, and 63% of this protein was retained after dialysis using a 14 kDa cut-off membrane. Similarly, Kichouh-Aiadi et al. [111] developed a two-step purification process integrating adsorption and cross-flow dialysis, which enabled the sequential recovery of phycobiliproteins and polysaccharides from Spirulina. These results demonstrate the effectiveness of cross-flow dialysis for separating high-value proteins while maintaining their functionality. This extraction and isolation approach is particularly valuable for the food industry because it preserves protein functionality while producing a soluble protein extract and a decolorized protein isolate.

On the other hand, ultrafiltration relies on pressure gradients (1–10 bar) across membranes (1–100 nm) to concentrate and purify proteins. Safi et al. [16] reported protein recovery rates of 24.8% (following enzymatic treatment combined with ultrafiltration/diafiltration) and 17.4% (after high-pressure homogenization combined with ultrafiltration/diafiltration) in *Nannochloropsis gaditana*. Compared with dialysis, ultrafiltration is faster and capable of processing larger volumes, making it more suitable for industrial scale applications. Its main advantages are scalability and processing speed, whereas its disadvantages include membrane fouling and potential losses in protein recovery during concentration and diafiltration.

By integrating precipitation with membrane-based purification techniques like dialysis or ultrafiltration, protein recovery can be optimized while achieving higher purity and maintaining functionality for diverse applications. For example, Andreeva et al. [74] compared a one-step ultrafiltration method with a two-step process combining ultrafiltration and HPLC. These methods achieved protein purities of 8.28% and 12.3%, respectively, for protein fractions with molecular weights above 50 kDa isolated from Spirulina and *Chlorella vulgaris*. These findings illustrate the potential benefit of combining membrane separation with a higher-resolution chromatographic step when greater protein purity is required, although the additional processing complexity and cost may limit its application at an industrial scale.

## 5. Protein Characterization

A detailed structural and functional characterization of microalgal protein extracts is essential to meet food industry standards and enhance their competitiveness with conventional protein sources. Although substantial progress has been made in protein quantification and amino acid (AA) profiling, significant knowledge gaps remain regarding higher-order protein structure, techno-functional properties, and allergenic potential. Therefore, advanced analytical approaches are crucial for optimizing extraction processes, ensuring product stability, assessing allergenicity, and fully realizing the potential of microalgal proteins as sustainable alternatives in food and beverage systems. This is especially important because different extraction and purification processes can affect not only protein recovery, but also the protein fraction that is ultimately measured as protein content. As highlighted in Section 3, the extraction method and cell wall architecture jointly determine which protein fractions are released and recovered; the following subsections examine how these upstream processing choices translate into measurable differences in the composition, structure characteristics, and functionality of microalgal proteins.

### 5.1. Protein Content and Amino Acid Profiling

Accurate quantification of protein in microalgal extracts is challenging because these extracts may contain non-protein nitrogenous compounds and because reported protein values depend strongly on the analytical method used. Conventional nitrogen-based methods, such as the Kjeldahl and Dumas methods, are widely applied but require species-specific nitrogen-to-protein conversion factors and may overestimate true protein content when non-protein nitrogen is present [112,113,114]. Colorimetric assays, such as the Bradford or Lowry methods, are also commonly used, but are sensitive to matrix interferences and can vary depending on sample preparation and extraction conditions [113,115]. Costa et al. [14] further demonstrated that protein quantification based on Kjeldahl nitrogen analysis and the sum of hydrolyzed amino acids may provide complementary information for Spirulina extracts. The study also applied amino-acid-specific correction factors to account for degradation and incomplete amino acid release during acid hydrolysis, highlighting the importance of analytical method selection when comparing protein content among whole biomass and purified extracts. In contrast, amino acid profiling provides a more direct assessment of protein composition and nutritional quality. This approach typically involves acid hydrolysis of proteins followed by chromatographic separation, most commonly using high-performance liquid chromatography (HPLC) with pre-column derivatization. Derivatization reagents such as fluorenylmethyloxycarbonyl chloride (FMOC) or *o*-phthalaldehyde (OPA) improve detection sensitivity, although they add extra analytical steps and may introduce recovery losses for some amino acid residues [116,117]. Appropriate corrections for hydrolysis-sensitive amino acids are particularly important when analyzing microalgae, where pigment-protein interactions and oxidative degradation can affect measured values. The main advantage of amino acid profiling is its higher specificity, but the disadvantage is that it is time-consuming and can underestimate hydrolysis-sensitive residues, such as cysteine, methionine, and tryptophan if appropriate correction factors are not applied.

Beyond total content, amino acid composition also influences functional and sensory properties. Recent findings by Chua et al. [22] suggest that chromatographic amino acid profiling can correlate not only with compositional quality but also with flavor-related attributes. Thus, compositional features that are nutritionally desirable may also create sensory challenges, including bitterness, discoloration, or off-odors, which in turn affect product acceptability and downstream processing strategies such as decolorization and deodorization. For example, the relatively high sulfur containing amino acid content of Spirulina (cysteine) and *Chlorella vulgaris* (methionine) may contribute to their distinct flavor profiles. This observation highlights another practical trade-off: compositional features that are nutritionally beneficial can also produce sensory challenges that affect product acceptability and downstream processing, including decolorization and deodorization. This link between composition and processing challenges leads directly to Section 3, where extraction and disruption methods are evaluated in terms of both protein recovery and preservation of protein quality.

Finally, composition-based physicochemical descriptors derived from AAIndex scales [118,119] can be used to estimate properties such as hydrophobicity, charge balance, and polarity, which influence protein folding, solubility, aggregation, and interaction potential. In this sense, amino acid profiling is not only a nutritional tool but also a useful predictor of functional behavior in food and biotechnological applications [120,121,122,123]. This reinforces the mechanistic relevance of composition–function relationships in protein-based applications, including fining agents and other food applications. This composition–function link is revisited in Section 5.5, where techno-functional performance is discussed directly.

Furthermore, hydrogen-bond donor and acceptor potentials can also be estimated using effective constants (α_eff and β_eff) derived from Hunter’s solvation model [124,125]. This approach provides a useful framework for relating amino-acid composition to protein solubility and interaction behavior, but it remains an indirect model and should therefore be complemented by experimental functional tests.

### 5.2. Proteomic Analysis

Sodium dodecyl sulfate-polyacrylamide gel electrophoresis (SDS-PAGE) is widely used to separate proteins based on molecular size [23]. Phycobiliproteins, including those found in Spirulina, have been identified as distinct subunits, such as the α-subunit of C-phycocyanin (17.6 kDa) and the β-subunit (18.4 kDa) [87]. Additionally, allophycocyanin subunits have been observed at 19.6 kDa and 17.7 kDa. Selvan et al. [126] determined the molecular weight of purified RuBisCO enzyme to be 54 kDa, while Mear et al. [127] identified small RuBisCO subunits of approximately 20 kDa and large subunits ranging from 22.6 to 40.4 kDa in *Tetraselmis chuii*.

However, SDS-PAGE alone has limitations in resolving proteins with closely overlapping molecular weights. To address this limitation, Suo et al. [128] combined SDS-PAGE with LC-MS/MS, enabling a more detailed analysis of Spirulina protein extracts. Their findings revealed a complex protein composition, with phycocyanin identified as the dominant protein. Techniques such as in-solution and in-gel digestion facilitate protein preparation for MS analysis, offering deeper insights into protein composition and functionality [24]. For instance, Ismaiel et al. [129] used SDS-PAGE to detect overall protein changes, followed by LC-MS/MS, which identified 77 specific proteins affected by environmental stress in Spirulina.

Smith et al. [130] analyzed proteins from *Tetraselmis* sp. using SDS-PAGE, detecting a band near 50 kDa. Further mass spectrometry analysis revealed additional proteins, including RuBisCO, ATP synthase (subunit β), and tubulin (α and β-subunits). Irvani et al. [131] investigated the proteome of Spirulina following aqueous protein extraction by high shear homogenization and pH shift. They found that acidic pH conditions resulted in the identification of a greater number of proteins. In addition, using the AllerCatPro database, they detected 12 putative allergenic proteins, the most abundant of which was the C-phycocyanin beta-subunit (P72508).

Guadalupi et al. [95] optimized an extraction protocol incorporating ultrasonication with a solution containing Tris(2-carboxyethyl) phosphine (TCEP), 2-chloroacetamide (CAA), ammonium bicarbonate (ABC), and sodium deoxycholate (SDC) at pH 8.5 for protein isolation from Spirulina and *Chlorella*. Subsequent in-solution digestion and proteomic analysis identified 1354 proteins in Spirulina and 771 proteins in *Chlorella*, each with more than 10% sequence coverage. These studies highlight the potential of advanced proteomic techniques to enhance the comprehensive characterization, functionality, and safety assessment of microalgal proteins.

Gao et al., [132] evaluated four protein extraction methods (direct buffer lysis, TCA acetone, phenol, and TCA-acetone/phenol) for proteomic analysis of *Chlorella vulgaris*. They identified 46 proteins, most of which were related to photosynthesis and localized in the chloroplast. Using SWATH based proteomics, a total of 760 proteins were identified, of which approximately 622 proteins overlapped with those detected by shotgun proteomics. This demonstrates the complementarity of these proteomic approaches and highlight the value of combining analytical approaches to achieve more comprehensive coverage of the microalgal proteome.

In a purified Spirulina protein extract obtained for wine-fining applications, LC–MS/MS analysis confirmed the predominance of phycobiliproteins. Allophycocyanin and phycocyanin subunits were among the main proteins identified, particularly in the 14–30 kDa electrophoretic region, while in-solution digestion enabled the identification of a broader set of 66 proteins [14]. These findings demonstrate the value of combining SDS-PAGE with in-gel and in-solution proteomics approaches to characterize both the dominant protein fraction and lower-abundance components.

### 5.3. Structural and Thermal Characterization

Structural and physicochemical characterization is essential for evaluating extract purity, conformational integrity, and suitability of microalgal protein extracts for food and beverage applications.

Fourier transform infrared (FTIR) spectroscopy provides complementary structural information by probing protein secondary structure and biochemical purity without extensive sample preparation. Attenuated total reflectance FTIR (ATR-FTIR) spectra (256 scans, 2 cm^−1^ resolution) enable the analysis of characteristic regions, including amide A (3300–3500 cm^−1^; N−H stretching), amide B (2900–3000 cm^−1^; C−H stretching), amide I (1600–1700 cm^−1^ C=O stretching), and amide II (1500–1560 cm^−1^; N−H bending and C−N stretching) [133,134,135].

The amide I band, primarily arising from C=O stretching vibrations of the peptide backbone, is highly sensitive to hydrogen bonding and molecular geometry, making it particularly suitable for determining secondary structure [136]. Second-derivative analysis and curve fitting allow its deconvolution into contributions from α-helix, β-sheet, turns, and random coils.

Characteristic bands at 1448 and 1418 cm^−1^ (C–H wagging) and the broad 1080–1030 cm^−1^ feature typical of phycobiliproteins [137,138,139,140] arise from overlapping bilin chromophore modes. These bands result from combined pyrrole-ring C–C/C–N stretching vibrations, along with methine-bridge and pyrrolic C–H out-of-plane deformations in the 1030–1090 cm^−1^ region [139,141]. FTIR also enables the indirect detection of residual lipids through the ester carbonyl stretching band around 1740 cm^−1^ [142] and polysaccharides via C–O and C–C stretching vibrations in the 1030–1100 cm^−1^ region [143]. Consequently, FTIR can provide useful information not only on protein conformation but also on the presence of non-protein components that may affect extract purity.

Thermal behavior assessed by thermogravimetric analysis (TGA) typically reveals a three-stage degradation profile [144,145]. The initial mass loss between 50 and 150 °C is attributed to the removal of physically adsorbed and weakly bound water associated with polar functional groups [146]. The second stage, with an onset around 260–270 °C, corresponds to the main degradation event involving peptide bond cleavage and amino acid side-chain decomposition [147]. At temperatures above 430 °C, a third stage is generally observed, associated with the slow degradation of carbon-rich residues and inorganic ash formation [148]. Costa et al. [14] used ATR–FTIR and thermogravimetric analysis to compare Spirulina protein extracts obtained under different extraction conditions. The extract produced at pH 7.0 with 1 M NaCl showed a protein profile consistent with phycobiliprotein-rich material and a lower residual mass at 900 °C than the extract produced at pH 9.0 without salt, indicating fewer non-protein residues and greater thermal stability under the conditions tested.

Together, FTIR and TGA provide complementary information on the molecular structure, purity, conformational characteristics, and thermal stability of microalgal protein extracts. These properties are particularly relevant when assessing their processing stability and suitability for incorporation into food and beverage formulations.

### 5.4. Isoelectric Points

The isoelectric points (pI) of microalgal proteins, including those derived from Spirulina, *Chlorella vulgaris*, and *Tetraselmis chuii*, have been reported within the acidic pH range, often around pH 2.6–3.0 [149,150,151,152,153]. At pH values near or below the isoelectric point, the reduction in net protein charge promotes protein aggregation, leading in minimal solubility [89]. This proximity of the isoelectric point to typical food-system pH values has direct practical implications for the techno-functional behavior discussed below, as low solubility near the isoelectric point can strongly limit emulsifying and foaming performance.

Conversely, moving the pH away from the pI increases the net protein charge and electrostatic repulsion, which generally promotes protein solubilization and can improve functional performance in food and beverage formulations.

### 5.5. Techno-Functional Properties and Digestibility

Microalgal proteins exhibit a range of techno-functional properties relevant to food formulation, including emulsifying, foaming, gelation, water holding, and oil binding capacities. Their performance depends on the species, protein composition, cell-disruption and extraction methods, pH, ionic strength, protein concentration, and degree of purification [154].

Emulsifying and foaming properties depend largely on the ability of proteins to adsorb at interfacial regions and form cohesive interfacial layers. *Tetraselmis* protein isolates have shown emulsifying and foaming properties under mildly acidic to neutral conditions, with favorable performance reported between pH 5 and 7 [150]. Similarly, *Arthrospira platensis* proteins have also shown emulsifying and foaming activity, although comparisons with soybean or egg proteins should be interpreted in relation to the specific assay conditions and protein concentrations used [155,156]. In the case of Spirulina sp., Pereira et al. [105] reported relatively high foaming capacity and stability, with foams remaining stable for up to 60 min. For *Tetraselmis* proteins, foam stability has been associated with the presence of limited amounts of charged polysaccharides, which may support the formation of a more stable interfacial network [150].

Processing conditions can substantially modify these functional properties. In a Spirulina protein preparation, ultrasound-assisted extraction increased the emulsifying stability index from 21.10 to 98.17 min, foaming capacity from 40.56 to 90.00%, foaming stability after 30 min from 35.85 to 79.17%, and water-holding capacity from 4.39 to 5.97 g/g, compared with extraction method without ultrasound treatment. The same treatment increased the reported in vitro protein digestibility from 16.08 to 33.44% [157]. These results indicate that ultrasound-assisted extraction may improve protein accessibility and selected techno-functional properties.

Water- and oil-holding capacities are influenced by protein hydration, surface charge, hydrophobicity, and the presence of residual non-protein components. *Arthrospira* proteins showed higher water-holding capacity under alkaline conditions [151]. *Tetraselmis* proteins displayed comparatively lower water-holding capacity in some assays [155]. Oil-binding activity was higher for *Arthrospira* protein isolates than for soybean protein and was further improved by ultrasonication, possibly because partial disruption of non-covalent interactions exposed hydrophobic regions able to interact with lipids [155]. These properties may be particularly relevant to emulsified foods, sauces, bakery products, meat analogues, and other formulations requiring moisture or lipid retention.

Drying conditions can also influence the functionality of *Chlorella vulgaris* proteins. Agitated thin-film drying improved protein solubility, water-holding capacity, gelation, and in vitro digestibility compared with some of the other drying treatments evaluated. However, this treatment also increased earthy off-flavor, indicating a potential trade-off between improved protein functionality and sensory acceptance [158].

Gelation depends on protein concentration, pH, ionic conditions, and structural modification. Calcium chloride improved gel formation in Spirulina protein systems [155]. However, extensive enzymatic hydrolysis may reduce gel-forming ability because smaller peptides generally have a lower capacity to form continuous protein networks [159]. High-pressure homogenization and bead milling reduced the minimum gelation concentrations of *Chlorella vulgaris* proteins, indicating improved gel-forming efficiency following cell disruption [160].

Digestibility is affected by cell-wall disruption, protein denaturation, extraction conditions, and the presence of non-protein components, such as polysaccharides, fibers, and pigments. In *Chlorella vulgaris*, high-pressure homogenization and bead milling were reported to improve in vitro digestibility, with values increasing from approximately 65% to above 80% under the conditions tested [160]. In Spirulina, ultrasound-assisted extraction resulted in higher reported in vitro digestibility than conventional extraction [157]. Overall, cell disruption and controlled processing can improve protein accessibility and selected techno-functional properties, but their effects depend on the treatment applied and the nutritional, technological, and sensory requirements of the final food product.

## 6. Potential Applications in the Food and Beverage Industry

The incorporation of microalgae into food products has gained increasing attention due to their ability to enhance nutritional value and improve functional properties. Both dried microalgal biomass and extracted proteins are being explored for their potential to transform conventional food and beverage formulations, offering sustainable and nutrient-dense alternatives [2].

Table 4 summarizes the various applications of microalgae in food products, highlighting their diverse benefits, including nutritional enhancement, the incorporation of functional ingredients, natural coloring, flavor enhancement, and their role in functional foods.

### 6.1. Microalgae Biomass Applications

The incorporation of microalgal biomass into food products can significantly enhance their nutritional, sensory, and functional properties. Microalgae can increase protein content, enrich products with essential amino acids and introduces bioactive compounds such as antioxidants, vitamins, and minerals. These components may contribute to the overall nutritional and health-promoting properties of the final product. The main advantage of using whole biomass is its relative simplicity of incorporation, since the biomass can often be added directly without the need of complete protein isolation; however, the drawback is that pigments, fibrous material, and characteristic flavors may negatively affect product appearance, texture, and consumer acceptance.

For example, the inclusion of Spirulina biomass in chocolate shake powder has been shown to enhance both protein and carbohydrate content, helping to meet the nutritional and energy needs of specific populations, such as the elderly [161]. In cookies, Spirulina incorporation has been reported to improve sensory characteristics, increase protein levels, and enhances phenolic content. Cookies containing 6% microalgal biomass, for example, exhibited the highest phenolic content (0.90 mg gallic acid equivalent/g), consistent with the phenolic content of the algae itself (19.0 mg gallic acid equivalent/g) [110]. Spirulina biomass is also used in sports nutrition drinks, where it increases protein levels and enhances the nutritional quality of the beverages [162].

Similarly, *Chlorella vulgaris* biomass has been incorporated into bread formulations, improving the rheological properties and texture of the final product, making it a valuable ingredient in baked goods [168]. It has also been incorporated into pasta and cookies as a natural coloring agent, aligning with clean-label and natural product trends [110].

The addition of various microalgal species to broccoli soup at concentrations ranging from 0.5 to 1.0% (*w*/*v*) has been shown to significantly increase polyphenol content (measured using the Folin-Ciocalteu method) and antioxidant capacity (assessed using the ferric ion reducing antioxidant power (FRAP) and DPPH radical scavenging assays), demonstrating the potential of microalgae for developing nutrient-rich meal options [171].

Furthermore, microalgal biomass has been incorporated into fermented products, where it may support the growth of beneficial bifidobacteria [169], and into yogurts, where it can enhance both antioxidant activity and protein content [170]. *Tetraselmis chuii* has also been studied for its ability to enhance the rheological properties of bread formulations, further emphasizing its functional benefits in the baking industry [172].

While these applications highlight the nutritional and functional advantages of microalgal biomass, sensory challenges, such as earthy or marine flavors, can affect consumer acceptance. To overcome these challenges, careful formulation strategies and flavor-masking techniques are essential to support wider market adoption. These sensory drawbacks represent one of the main barriers to the commercialization of whole microalgal biomass, especially when biomass is added at levels high enough to affect the texture and color of the final product.

### 6.2. Protein Extract Applications

The extraction of proteins with valuable functional and technological properties from microalgae presents a significant opportunity for the food industry. These properties, such as emulsifying, foaming, and gelling capacities, can enhance the quality, stability, and functionality of a wide range of food products. Compared with whole biomass, purified protein extracts are more suitable when the main goal is to provide specific functionality rather than pigmentation or whole-food enrichment.

For instance, protein extracts from *Chlorella vulgaris* have demonstrated excellent emulsifying properties, making them ideal for enhancing the texture and stability of various food formulations [96]. Similarly, proteins from *Tetraselmis* species have been shown to form stable emulsions, indicating their potential for use in emulsified food products [150].

Spirulina protein extracts exhibit a range of functionalities, including foaming and gelling capabilities, which are especially valuable in food formulation [151]. For example, Spirulina proteins have been used as emulsifying and foaming agents to stabilize fluid interfaces and enhance the sensory properties of food systems [163]. These properties depend strongly on protein structure, surface charge, and solubility, which means different extraction conditions can produce different functional outcomes even from the same species.

In baked goods, Spirulina powder has been incorporated into biscuits to increase protein content as well as antioxidant potential, both of which rise in proportion to the amount of Spirulina added [164]. In addition, Spirulina proteins have been investigated for their role in cheese packaging, where they enhance the performance of whey protein-based edible films, offering biodegradable and innovative packaging solutions [165]. Moreover, Spirulina protein extracts have been incorporated into beverages, such as “Pro-Tea,” which is fortified with antioxidants and protein to increase its nutritional value [166]. They have also been incorporated into yogurt formulations, where phycocyanin contributes to enhanced nutritional and functional properties [167].

Although these examples highlight the versatility and technological potential of microalgal proteins in food applications, research into their practical use remains relatively limited. This gap presents a clear opportunity for further development, especially in emerging sectors such as plant-based or allergen-free foods. Further research is needed to compare the functional performance of proteins from different microalgal species and to determine whether the benefits observed at laboratory scale can be maintained in industrial-scale formulations. In winemaking, microalgal protein extracts have potential as sustainable fining agents. Costa et al. [14] provided the first comprehensive evaluation of a purified Spirulina protein extract as a wine fining agent. The extract was produced by bead milling at pH 7.0 in the presence of 1 M NaCl, followed by ethanol precipitation, and contained approximately 63% protein, with a protein recovery of approximately 25%. At a dose of 50 g/hL, the *Spirulina* extract removed 81% of total proanthocyanidins in a white wine model solution, showing performance comparable to that of egg albumin, pea protein, and patatin, although it was less effective than gelatin and casein. In a red wine model solution, the extract removed 30% of total proanthocyanidins, exceeding the performance of the commercial fining agents tested (approximately 3–19%). These differences demonstrate that fining efficacy is strongly dependent on wine-matrix composition and on the structural characteristics of both the proteins and proanthocyanidins [14]. However, purified microalgal protein extracts are not currently commercially available, and protein extracts from these microalgae have not yet been directly evaluated as wine fining agents in real wine. Noriega-Domínguez et al. [173] assessed commercial Spirulina biomass capsules (65% protein) as a fining agent, referring to them as a “protein extract”. However, this approach did not involve the actual laboratory isolation or purification of Spirulina proteins. The Spirulina biomass capsules exhibited complete water solubility, an isoelectric point of 2.8 (close to the wine pH) and a low surface charge density (22.1 ± 10^−6^). Their molecular masses ranged from less than 6.5 to 191.5 kDa, with two major bands at 21.0 and 23.4 kDa. Spirulina outperformed gelatin in reducing turbidity and producing more compact sediment. However, sensory evaluations revealed the presence of some off-flavors and odors.

Furthermore, microalgae-based fining agents may offer a more sustainable and environmentally friendly alternative to traditional animal-derived proteins, with significantly lower requirements for land and water use, and a potentially smaller environmental footprint [13,163].

Overall, the incorporation of microalgal protein extracts into food and beverage systems represents a promising avenue for innovation. Their nutritional and techno-functional properties, combined with the potential for sustainable production, could support the development of novel products that respond to the growing consumer demand for sustainable, functional, and non-animal-derived protein ingredients.

## 7. Regulations and Safety of Microalgae

The consumption and commercialization of microalgae-based products are regulated through a framework designed to ensure their safety, quality, transparency, and compliance with applicable standards. In the United States, microalgae-derived ingredients may be marketed through the Generally Recognized as Safe (GRAS) pathway when qualified experts determine that their intended use is safe under the specified conditions of use. In the European Union, microalgae-based products that were not consumed to a significant degree before May 1997 are regulated as novel foods under Regulation (EU) 2015/2283 [6]. The European Commission is responsible for authorization, while the European Food Safety Authority (EFSA) conducts the scientific safety assessment. This centralised framework means that regulatory status depends not only on the species itself, but also on the product format, production process, and intended use. Consequently, even well-established microalgal species may require product-specific assessment when introduced in new forms or as concentrated ingredients.

For novel food applications, EFSA requires a comprehensive dossier covering the identity of the food, production process, compositional data, specifications, proposed uses and use levels, anticipated intake, history of use, absorption, distribution, metabolism, and excretion (ADME) data, nutritional information, toxicological information, and allergenicity. This is particularly relevant for protein-rich ingredients and protein isolates, because concentrated fractions may have different exposure patterns and safety profiles from those of whole biomass products. EFSA guidance also considers allergenicity an important component of the safety assessment of protein-containing novel foods, particularly when the source organism is related to known allergenic sources or when the product contains proteins with possible cross-reactivity. Therefore, labeling should clearly inform consumers about ingredients and any relevant allergenic substances, particularly when allergenic proteins or allergen-like sequences have been identified.

For example, Spirulina and *Chlorella vulgaris* are authorized for use as food ingredients within the European Union, as stipulated in Regulation (EU) 2015/2283 [6]. Since these species were widely consumed prior to 1997, they are exempt from the EU’s Novel Food Regulation [17]. At present, the majority of commercially available microalgae-based food products are derived from these two established strains [171]. Additionally, *Tetraselmis chuii* has been classified as safe under the European Commission’s “no toxins known” designation [174]. However, authorisation depends on the specific product, production process, and intended use, rather than on species status alone.

The industrial-scale cultivation of microalgae such as *Arthrospira* sp. (Spirulina) and *Chlorella* sp. enables their widespread availability in various formats, including tablets, capsules, liquids, and nutritional supplements [12]. These microalgae are also widely incorporated into food formulations due to their high nutritional value, presence of bioactive compounds, and potential health benefits. For novel food applications, applicants must provide detailed information on identity, composition, production process, proposed uses, intake estimates, nutritional impact, toxicological data, and allergenicity. In particular, protein-rich ingredients and protein isolates may require specific attention because concentrated fractions can differ from whole biomass in terms of exposure pattern, composition, and safety profile.

Safety assessment of microalgae should also consider allergenicity at both the species and protein levels. Certain Spirulina-derived proteins may have allergenic potential, as evidenced by a reported case of anaphylaxis in a 14-year-old triggered by the protein P72508 (Art pl beta-phycocyanin) [175]. Hamzelou et al. [38] identified putative allergenic proteins in food-grade *Nannochloropsis oculata* extracts with high similarity to known food allergens. This means that species generally regarded as safe in general can still contain individual proteins with allergenic potential, so safety must be evaluated at both the species and protein levels. Accordingly, allergenicity should be considered a default issue in safety assessment, particularly for protein-containing novel foods, especially when the source organism is related to known allergens or when cross-reactive proteins may be present. To improve allergen detection, Bianco et al. [176] proposed a novel method combining proteomic analysis with in silico sequence homology prediction. Notably, to date, no proteins from *Arthrospira platensis* have been officially recognized in the systematic allergen nomenclature approved by the Allergen Nomenclature Subcommittee of the World Health Organization (WHO) and the International Union of Immunological Societies (IUIS) [177]. Furthermore, acute and chronic toxicity studies suggest that the consumption of approved microalgae does not pose significant health risks [61].

Transparent labelling remains essential to informing consumers about ingredients, nutritional value, potential allergens, and product origin. Although there are currently no regulatory requirements mandating explicit allergen labelling for microalgae-based products [3], consumers should still be adequately informed whenever a product contains ingredients with potential allergenic relevance. This transparency can help prevent misleading claims such as “non-allergenic” or “sustainable,” and is essential for building consumer trust. It is also particularly important for protein isolates and other concentrated ingredients, since they may present different regulatory and safety considerations than whole-cell products.

The production and commercialization of bioactive microalgal extracts still face several challenges. Key barriers include high production costs, the need for appropriate strain selection, optimization of cultivation conditions, and the development of efficient, scalable extraction and purification methods. In addition, the regulatory burden can slow market entry, especially for innovative products such as proteins produced by precision fermentation. In the European Union, such products are generally assessed within the novel food framework and may also raise additional questions related to GM legislation, depending on the production system. Industry reports indicate that lengthy and uncertain authorisation timelines, limited pre-submission guidance, and the requirement to test the exact product intended for market can create substantial financial and operational risk for companies. For this reason, regulatory complexity remains one of the principal barriers to the wider adoption of novel proteins, including microalgae-derived proteins and precision-fermented alternatives.

## 8. Conclusions and Future Perspectives

Microalgae and plant-derived proteins are widely recognized for their significant contribution to the United Nations 2030 Agenda for Sustainable Development, particularly in advancing food security, social development, and environmental protection. The growing importance of microalgal proteins in promoting sustainable food systems is increasingly acknowledged, owing to their ability to address key global challenges. These include environmental sustainability (as photosynthetic organisms), food security (owing to their relatively low allergenic potential), dietary diversification (as suitable options for vegan, vegetarian, and natural diets), and unique competitive advantages over traditional protein sources (such as high biomass productivity and reduced resource requirements).

Compared with livestock farming, microalgae cultivation can have a significantly smaller environmental footprint and can provide a stable, reliable source of protein throughout the year. Moreover, localized microalgae production systems hold great promises for enhancing socioeconomic opportunities, especially in regions with limited agricultural land and scarce natural resources.

Species such as Spirulina, *Chlorella vulgaris* and *Tetraselmis chuii* offer high protein contents and provide many of the essential amino acids required in the human diet, making them valuable alternatives to conventional protein sources [178]. However, it is important to note that histidine is often identified as the most limiting essential amino acid in these microalgal species [73].

To facilitate the full integration of microalgal proteins into food systems, several critical research areas and challenges must be addressed:

- Expanding the diversity of protein-producing species: Future research should focus on broadening the range of microalgae used for protein production, including species such as *Scenedesmus obliquus* [39], *Scenedesmus rubescens*, *Nannochloropsis oculata* [38], and *Chlorococcum amblystomatis*. These species are known for their high protein content and balanced amino acid profiles. Incorporating macroalgae may also increase overall protein output and enhance product diversity.
- Generating scale-up data for extraction and purification: Future work should move beyond laboratory-scale demonstrations and provide quantitative pilot- and industrial-scale data for key processes, including high-pressure homogenization, ultrasound-assisted extraction, bead milling, and combined mechanical–enzymatic or chemical treatments. These studies should evaluate process performance, operating costs, energy demand, protein recovery, scalability, and preservation of protein functionality using standardized criteria and comparable conditions. The development of environmentally friendly, efficient, and scalable techniques for cell disruption, protein extraction, and purification is critical. Industrial adoption of microalgal proteins will depend on the economic viability and environmental sustainability of these processes. Bead milling, ultrasonication, ammonium sulfate fractional precipitation, and dialysis are among the most used methods for microalgae protein extraction and purification.
- Developing systematic criteria for extraction-method selection: The data generated at pilot and industrial scales should support decision frameworks that link method selection to the microalgal species, cell-wall structure, intended food application, and required product properties. HPH and combined methods may be suitable when high protein recovery is the priority, although they generally involve higher energy demand and process complexity. Bead milling is preferable for scalable and non-thermal processing. UAE may be advantageous for rapid extraction, while enzymatic lysis is more appropriate when mild processing conditions and protein functionality must be preserved.
- Ensuring regulatory compliance and safety: Achieving widespread market acceptance will require microalgal proteins to comply with international food safety regulations, obtain appropriate regulatory approvals, and meet global food safety standards. As discussed in Section 7, allergenicity data for microalgal proteins remain incomplete, with only isolated case reports and in silico predictions available for some species. Focused research combining proteomics, sequence-homology screening, and targeted immunological or clinical studies is needed to identify potential allergens in microalgal protein isolates and precision-fermented products, thereby supporting transparent labeling and robust risk assessment.
- Systematic techno-functional characterization and sensory optimization: To gain consumer acceptance, microalgal proteins must meet industry expectations regarding sensory quality (taste, color, texture) and technological functionality (e.g., emulsification, gelling, foaming, water- and oil-holding capacity, digestibility). Existing data, summarized in Section 5.5, are fragmented and often limited to laboratory model systems. Future work should use standardized protocols to compare the techno-functional properties of protein fractions from different species and extraction routes in representative food matrices (such as beverages, meat analogues, and dairy alternatives), while explicitly linking extraction conditions to functional performance and sensory impact.

In addition, microalgal proteins must offer functional and technological properties comparable to or superior to those of traditional protein sources while remaining economically viable. Regulatory and economic barriers identified in Section 7 also highlight the need to improve process robustness, reduce batch-to-batch variability, and generate realistic exposure and safety data for protein isolates and precision-fermented microalgal proteins. These developments are essential to support regulatory authorization and market entry. A key challenge is ensuring that microalgal proteins function effectively as food ingredients while maintaining or improving the sensory and nutritional quality of the final product.

The future development of this field will depend on the simultaneous improvement of technical performance, production economics, regulatory compliance, and consumer acceptance.

Encouragingly, advances in biotechnology and rising consumer interest in sustainable food alternatives indicate a bright future for microalgal proteins. By addressing current technological, economic, and regulatory challenges and expanding their range of applications, these proteins are well-positioned to play a transformative role in the future of sustainable food production. If these challenges are effectively addressed, microalgal proteins could move from niche ingredients to mainstream components of next-generation food systems.

## Figures and Tables

**Table 1:** Amino acid profiles of microalgal biomass from Spirulina, *Chlorella vulgaris*, and *Tetraselmis chuii*, expressed in g/100 g protein, together with the FAO indispensable amino acid scoring pattern of older children, adolescents, and adults.

Amino Acids	Spirulina [69]	Spirulina [10]	*Chlorella vulgaris* [37]	*Chlorella vulgaris* [70]	*Tetraselmis chuii* [37]	*Tetraselmis chuii* [70]	FAO [71]
His	1.1 ± 0.0	1.8 ± 0.1	0.6 ± 0.0	1.1 ± 0.1	0.6 ± 0.0	0.8 ± 0.1	1.6
Lys	3.4 ± 0.0	4.9 ± 0.0	3.2 ± 0.1	3.9 ± 0.3	1.9 ± 0.0	3.1 ± 0.4	4.8
Leu	6.2 ± 0.2	9.3 ± 0.0	1.9 ± 0.1	4.5 ± 0.2	2.3 ± 0.1	4.9 ± 0.5	6.1
Ile	3.6 ± 0.2	5.9 ± 0.0	0.9 ± 0.1	1.8 ± 0.0	1.2 ± 0.1	2.2 ± 0.2	3.0
Met	1.7 ± 0.1	2.5 ± 0.0	0.7 ± 0.1	1.5 ± 0.1	0.8 ± 0.1	1.3 ± 0.2	-
Met + Cys	-	-	-	-	-	-	2.3
Phe	3.3 ± 0.1	4.9 ± 0.0	0.9 ± 0.0	2.8 ± 0.2	1.6 ± 0.1	3.5 ± 0.6	-
Phe + Tyr	-	-	-	-	-	-	4.1
Thr	3.3 ± 0.1	5.1 ± 0.0	1.2 ± 0.0	2.6 ± 0.1	1.7 ± 0.0	3.0 ± 0.4	2.5
Val	4.2 ± 0.1	6.3 ± 0.0	1.5 ± 0.1	2.8 ± 0.1	1.8 ± 0.1	3.3 ± 0.4	4.0
Ala	5.0 ± 0.1	7.6 ± 0.0	2.4 ± 0.1	4.4 ± 0.3	2.5 ± 0.1	4.4 ± 0.6	-
Asp	6.3 ± 0.2	10.2 ± 0.0	3.7 ± 0.1	5.2 ± 0.4	5.2 ± 0.1	6.5 ± 0.9	-
Glu	8.5 ± 0.2	14.6 ± 0.1	3.5 ± 0.1	6.3 ± 0.4	4.8 ± 0.1	8.6 ± 1.0	-
Gly	3.4 ± 0.1	4.5 ± 0.0	1.9 ± 0.1	2.8 ± 0.2	2.1 ± 0.0	3.4 ± 0.5	-
Pro	2.5 ± 0.0	4.1 ± 0.1	2.1 ± 0.1	2.6 ± 0.2	2.0 ± 0.0	3.3 ± 0.5	-
Ser	N/A	5.1 ± 0.0	1.2 ± 0.0	2.4 ± 0.2	1.2 ± 0.1	3.0 ± 0.4	-
Tyr	3.1 ± 0.1	4.3 ± 0.0	0.7 ± 0.0	2.2 ± 0.1	1.0 ± 0.0	2.0 ± 0.3	-
Cys	0.6 ± 0.0	0.8 ± 0.0	0.3 ± 0.0	0.7 ± 0.0	0.5 ± 0.0	1.2 ± 0.1	-
Arg	4.5 ± 0.1	6.6 ± 0.1	2.7 ± 0.1	3.1 ± 0.2	3.8 ± 0.0	3.5 ± 0.4	-

His: histidine; Lys: lysine; Leu: leucine; Ile: isoleucine; Met: methionine; Phe: phenylalanine; Thr: threonine; Val: valine; Ala: alanine; Asp: aspartic acid; Glu: glutamic acid; Gly: glycine; Pro: proline; Ser: serine; Tyr: tyrosine; Cys: cysteine; Arg: arginine. N/A: not available.

**Table 2:** Summary of cell disruption methods used for protein extraction.

Methods	Description	Protein Recovery	Energy Consumption	Scalability	Preservation of Protein Functionality	Best Suited for	References
High-Pressure Homogenization	Applies high pressure to disrupt cells through hydrodynamic cavitation.	High	High	Moderate–high	Moderate	High recovery and rapid processing	[15,16,18,45,76,78,79,80]
Ultrasonic-Assisted Extraction	Uses ultrasonic waves to create cavitation, disrupting cell walls.	Moderate–high	High	Moderate	Moderate	Rapid extraction with reduced solvent use	[3,17,45,77,81,82,83,84,85,86]
Bead Milling	Disrupts cells by agitating beads in a milling chamber.	Moderate	Low–moderate	High	High	Scalable, non-thermal processing	[3,9,14,15,45,75,87,88,89]
Enzymatic Lysis	Uses enzymes to selectively break down cell walls, enhancing protein release.	Moderate–high	Low–moderate	Low–moderate	High	Mild processing and preservation of functionality	[11,15,19,20,77,86,90,91,92]
Chemical Treatment	Employ salts or organic solvents to modify pH and facilitate cell lysis.	Moderate	Low–moderate	Moderate	Low–moderate	Simple and low-cost processing	[86,93]
Combined Methods	Utilizes a combination of mechanical and enzymatic or chemical techniques to enhance extraction.	High	Moderate–high	Low–moderate	Moderate–high	Maximum recovery through optimized processing	[94,95]

**Table 3:** Summary of extraction methods reported in the literature for different algal species.

Disruption Cell	Microalgae Species	Conditions	Quantification Method	Protein Content in Biomass (% dw)	Protein Content in Extract (*w*/*w*).	References
High-pressure homogenization	Spirulina	*p* = 270 MPa. np = 2	Lowry	53.5 ± 1.1	78.0 ± 2.8	[45]
	Spirulina	*p* = 30–90 MPa	Kjeldahl	57.9 ± 0.5	-	[18]
	*Chlorella vulgaris*	*p* = 270 MPa. np = 2	Lowry	49.6 ± 1.0	52.8 ± 0.6	[45]
	*Chlorella vulgaris*	*p* = 150 MPa. np = 5	Lowry	-	33.0	[78]
	*Chlorella vulgaris*	*p* = 103 MPa. np = 5	Bradford	-	75.6 ± 3.8	[79]
	*Chlorella vulgaris*	*p* = 270 MPa. np = 2. pH 12	Lowry	53.0	52.0 ± 3.0	[96]
	*Tetraselmis chuii*	*p* = 30–90 MPa	Kjeldahl	15.5 ± 0.5	-	[18]
	*Tetraselmis suecica*	*p* = 30 MPa np = 2	Kjeldahl	23.1 ± 0.3	61.9 ± 3.9	[80]
Ultrasonic-assisted extraction	Spirulina	Probe (20 kHz. 13 mm) for 30 min—5 s of ultrasound and 15 s of pause	Lowry	53.5 ± 1.1	47.1 ± 0.9	[45]
	Spirulina	Phosphate-buffered saline and ultrasound probe (400 W and 30 kHz)	Kjeldahl	62.7	52.23 ± 0.5	[97]
	*Chlorella vulgaris*	Probe (20 kHz. 13 mm) for 30 min—5 s of ultrasound and 15 s of pause	Lowry	49.6 ± 1.0	18.1 ± 0.0	[45]
	*Chlorella vulgaris*	Ultrasonic reactor, 0–3 bar—100, 200, and 300 W at low frequency of 12 kHz	Kjeldahl	58.0 ± 1.0	-	[98]
	*Chlorella vulgaris*	Probe (35 to 130 kHz), combined with enzymes, solvents and sequential extraction	Nitrogen analyser	24.3 ± 0.0	-	[99]
	*Chlorella vulgaris*	30 min contact time	Bradford	-	76.6 ± 1.0	[79]
	*Nannochloropsis* sp. *P. tricornutum,* and *P. Kessler*	24 kHz	Bradford	-	-	[77]
Bead milling	Spirulina	5 min contact time	Lowry	53.5 ± 1.1	35.0 ± 1.2	[45]
	Spirulina	4 cycles of 25 s at 30 Hz	Lowry	-	46.1 ± 4.4	[87]
	Spirulina	pH 7.0; 1 M NaCl	HPLC	54.6 ± 1.7	63.2 ± 2.1	[14]
	*Chlorella vulgaris*	5 min contact time	Lowry	49.6 ± 1.0	9.0 ± 0.1	[45]
	*Chlorella vulgaris*	15 min contact time	Bradford	-	75.5 ± 0.5	[79]
	*Chlorella vulgaris*	Constant agitation speed of 2039 rpm. and enzymatic hydrolysis	Lowry	40.0	68.0	[75]
	*Tetraselmis chuii*	30 min contact time	Dumas	36.0	24.0	[89]
	*Tetraselmis suecica*	Constant agitation speed of 2039 rpm	Lowry	54.0 ± 4.0	50.4	[9]

Note: *p*: pression-; np = number of passes.

**Table 4:** Applications of microalgae in food products including Spirulina, *Chlorella vulgaris*, and *Tetraselmis chuii*.

Microalgae Source	Type	Food Products	Applications	References
Spirulina	Biomass	Chocolate shake powder	Source of protein and carbohydrates, contributing to the energy and nutrient requirements of the elderly	[161]
	Biomass	Cookies	Enhance the sensorial characteristics, increase the protein and phenolic content	[110]
	Biomass	Sports nutrition drinks	Boosts protein levels and nutritional quality	[162]
	Protein Extract	Emulsions and foams agent	Suitable for food formulations	[151]
	Protein Extract	Emulsions and foams agent	Alternative to stabilize fluid interfaces, emulsions, and foams	[163]
	Phycocyanin extract	Biscuit	Increase fibre and protein content	[164]
	Protein Extract	Cheese packaging	Whey protein-based edible films	[165]
	Protein Extract	Beverage—“Pro-Tea”	Enriched with antioxidants and protein for nutrition care	[166]
	Protein Extract	Yogurt	A phycocyanin powder used to enhanced nutritional and functional characteristics	[167]
	Protein Extract	Wine fining in model wine	Removal of proanthocyanidins; alternative to commercial protein fining agents.	[14]
*Chlorella vulgaris*	Biomass	Bread dough	Impacts rheological characteristics	[168]
	Biomass	Fermented products	Exerts beneficial effects on bifidobacteria cultures	[169]
	Biomass	Yogurts	Enriches antioxidant activity and protein content	[170]
	Biomass	Broccoli soup	Increases polyphenol content and enhances antioxidant capacity	[171]
	Protein Extract	Emulsion and a stability agent	Provides emulsifying capacity for improved texture and stability in food formulations	[45]
*Tetraselmis chuii*	Protein Extract	Bread	Enhance the rheological characteristics	[172]
	Protein Extract	Emulsion properties	Forms stable emulsions	[150]

## Data Availability

The original contributions presented in this study are included in the article. Further inquiries can be directed to the corresponding author.
